# Phase separation and DAXX redistribution contribute to LANA nuclear body and KSHV genome dynamics during latency and reactivation

**DOI:** 10.1371/journal.ppat.1009231

**Published:** 2021-01-20

**Authors:** Olga Vladimirova, Alessandra De Leo, Zhong Deng, Andreas Wiedmer, James Hayden, Paul M. Lieberman

**Affiliations:** 1 The Wistar Institute, Philadelphia, United States of America; 2 Department of Immunology, H. Lee Moffit Cancer and Research Center, Tampa Florida, United States of America; University of Southern California, UNITED STATES

## Abstract

Liquid-liquid phase separation (LLPS) can drive formation of diverse and essential macromolecular structures, including those specified by viruses. Kaposi’s Sarcoma-Associated Herpesvirus (KSHV) genomes associate with the viral encoded Latency-Associated Nuclear Antigen (LANA) to form stable nuclear bodies (NBs) during latent infection. Here, we show that LANA-NB formation and KSHV genome conformation involves LLPS. Using LLPS disrupting solvents, we show that LANA-NBs are partially disrupted, while DAXX and PML foci are highly resistant. LLPS disruption altered the LANA-dependent KSHV chromosome conformation but did not stimulate lytic reactivation. We found that LANA-NBs undergo major morphological transformation during KSHV lytic reactivation to form LANA-associated replication compartments encompassing KSHV DNA. DAXX colocalizes with the LANA-NBs during latency but is evicted from the LANA-associated lytic replication compartments. These findings indicate the LANA-NBs are dynamic super-molecular nuclear structures that partly depend on LLPS and undergo morphological transitions corresponding to the different modes of viral replication.

## Author summary

During latent infection, gamma-herpesvirus genomes are maintained as extrachromosomal circular DNA, referred to as episomes, by dedicated viral-encoded episome maintenance proteins. KSHV-encoded LANA maintains viral episomes through binding as an oligomeric protein to repetitive DNA elements in the viral terminal repeats (TRs). Viral episomes can be visualized as LANA-associated nuclear bodies (LANA-NBs). Here, we show that LANA-NBs utilize mechanisms of self-assembly through liquid-liquid phase separation (LLPS) to build dynamic structures that change during cell cycle and viral life cycle. We find that DAXX is a component of the latent phase LANA-NBs, but is evicted during the transition to lytic replication where LANA remains associated with KSHV DNA to form a ring-like replication compartment.

## Introduction

Kaposi’s Sarcoma-Associated Herpesvirus (KSHV)/Human Herpesvirus 8 (HHV8) is the causative agent of Kaposi’s Sarcoma (KS), Primary Effusion Lymphoma (PEL), and Multicentric Castleman Disease (MCD) [1,2]. KSHV pathogenesis is associated with the latent infection where the viral genome persists as a multi-copy circular genome, referred to as the episome, in the nucleus of an infected cell [3,4]. The viral episomes are subject to chromatin assembly and epigenetic regulation that restrict viral gene expression to only a few viral genes, yet remain poised for rapid reactivation to complete the viral life cycle [5–7]. During latency, viral transcription and replication depend on host machinery. In addition, viral episomes are maintained at stable copy numbers in proliferative cells indicating they have dedicated mechanisms to ensure faithful segregation during cell division.

The KSHV encoded latency associated nuclear antigen (LANA) is a key regulator of KSHV latency [4,8]. LANA binds directly to the KSHV terminal repeat (TR) through its DNA binding domain (DBD) that has structural similarity to episome maintenance proteins of EBV EBNA1 and HPV E2 [9,10]. LANA DNA binding is essential for viral episome maintenance and initiation of DNA replication at the viral TR [11]. LANA can bind to host chromosome sites and regulate host transcription [12–15]. LANA can also bind directly and indirectly to other regions of the viral genome, including regulatory elements upstream of the lytic activator RTA [16,17], and autoregulatory elements upstream of the ORF73 transcript encoding LANA [18]. Structural studies of the LANA DBD revealed formation of numerous oligomeric structures in X-ray crystallography and extended filaments by electron microscopy [10,19,20]. The oligomerization of LANA was shown to be important for several functions of LANA, including episome maintenance and autoregulation [18,20]. LANA oligomerization is also important for 3D chromosome conformation of the KSHV genome and responsible for a DNA-DNA loop interaction between the TR and the LANA promoter region [18].

LANA in association with KSHV episomes forms large nuclear structures, termed LANA speckles or LANA Nuclear Bodies (NBs) [11]. These LANA-NBs colocalize with several cellular proteins, including the histone H3.3 chaperone DAXX, the Polycomb-associated histone H3K27me3 methylase EZH2, histone H3K27me3, and the origin recognition complex protein ORC2 [18]. LANA-NBs do not colocalize with the promyelocytic leukemia (PML) protein that is known to form PML-nuclear bodies, indicating that LANA-NBs are distinct from PML-NBs [18]. LANA-NBs were found to be highly stable in number and organization throughout the cell cycle. It is well-established that LANA can interact with histone H2A/H2B core histones through its N-terminal tethering domain, and that this interaction is essential for episome maintenance and chromosome attachment [21]. LANA can also interact with numerous other proteins, including chromatin regulatory factors [3,22,23]. How these various interactions are organized into a stable 3D structure that allows for regulated transcription and replication remains poorly understood.

The physical properties that enable higher-ordered structures to self-assemble have been investigated for numerous biological systems. Membrane-less biomolecular condensates that form through phase separation chemistry have been implicated in many cellular and virological processes [24–26]. For example, higher ordered heterochromatin depends on the liquid-liquid phase separation (LLPS) properties of HP1, which can facilitate the condensation of chromatin domains through multivalent interactions including recognition of methylated histone H3K9me3 [27]. Transcriptional super enhancers form through the condensate properties of multivalent proteins including members of the BRD family that recognize acetylated histones [28,29]. Post-translational modifications of the heptad repeats in RNA polymerase C-terminal tail nucleate condensates that regulate RNA splicing [30]. In addition, many of these membrane-less structures depend on nucleic acid templates, such RNA dependence of paraspeckles and Cajal bodies [31,32], and repetitive ribosomal DNA for the formation of nucleoli [33]. Viral specific structures are likely to be required to protect and sequester viral genomes away from host genomes, as well as to provide sufficient concentration of components for successful viral replication [34,35] (reviewed in [36]). We have previously investigated LANA NBs and shown that these structures depend, in part, on the oligomerization of the LANA DBD and colocalize with histone H3.3 chaperone DAXX during latency [18]. Here we investigate whether LANA NBs are also dependent on LLPS and how these structures may change during the viral life cycle.

## Results

### LANA nuclear bodies are sensitive to LLPS disrupting agents

**Fig 1:**
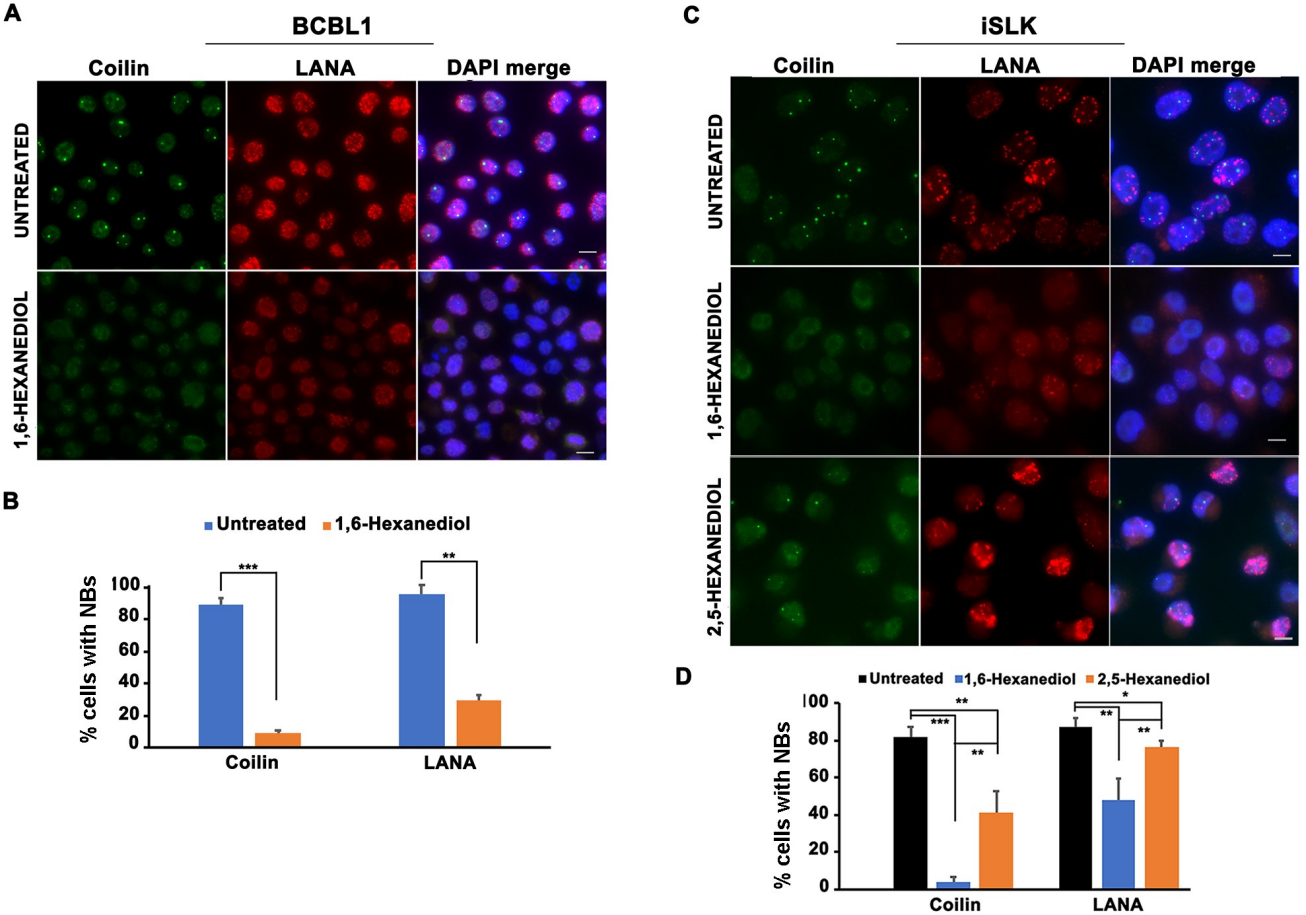
LANA-NBs are partially sensitive to LLPS disrupting agents. **A**. BCBL1 cells untreated or treated for 10 min with 3.5% 1,6-Hexanediol were imaged by IF for Coilin (green), LANA (red) or DAPI (blue). Scale bar = 10 μm. **B**. BCBL1 cells treated as in panel A were quantified for >2 coilin or >4 LANA NBs. The bar graph represents means ± s.d, ** p value < 0.01, *** p value < 0.001 using two-tailed student t-test. **C**. iSLK RFP-LANA cells untreated or treated for 10 min with 3.5% 1, 6-Hexanediol or 2,5-Hexanediol were imaged by IF for Coilin (green), RFP-LANA (red) or DAPI (blue). Scale bar = 10 μm. **D**. iSLK RFP-LANA cells treated as in panel C were quantified for >2 coilin or >4 LANA-NBs. The bar graph represents means ± s.d, * p value < 0.05, ** p value < 0.005, *** p value < 0.001 using two-tailed student t-test.

Biological structures that depend on LLPS are sensitive to 1,6-Hexanediol (HD), but less sensitive to 2,5-Hexanediol [33]. To determine whether LANA-NBs were dependent on LLPS dynamics, we first treated KSHV positive pleural effusion lymphoma cell line BCLB1 with 1,6-HD assayed for dispersion of LANA-NBs (Fig 1A and 1B and S1A–S1C Fig). We determined that treatment with 3.0–3.5% 1,6-HD for at least 10 min was sufficient to disrupt coilin-associated Cajal bodies (S1 Fig), consistent with published reports for other cell lines [33]. Cajal bodies function in the assembly of ribonucleoprotein complexes, including spliceosomal snRNPs and telomerase, and their ability to assemble through liquid-liquid phase transition has been correlated with these biological activities [37]. We observed that BCBL1 and iSKL cells typically have ~2 Cajal bodies and >4 LANA-NBs in the majority of cells. We found that 1,6-HD reduced the number of cells with 2 coilin NBs to <10%, while cells with >4 LANA-NBs were reduced to ~30%. LANA-NBs had similar kinetics of disruption and recovery as did coilin foci in response to 1,6-HD (S1 Fig). To further characterize LANA-NBs and obtain higher quality imaging, we utilized the previously characterized adherent cell line iSLK carrying KSHV Bac16 expressing RFP-LANA [18]. We treated iSLK RFP-LANA cells with 3.5% 1,6-HD or the less disruptive control 2,5-HD for 10 min and assayed by IF for LANA-NB or coilin foci. We found that 2,5-HD treatment resulted in ~41% of cells with >2 coilin foci compared to <5% in 1,6-HD treated cells (Fig 1D). Similarly, 2,5-HD treatment resulted in ~78% of cells with >4 LANA-NBs, compared to ~48% in 1,6-HD treated cells. Taken together, these findings indicate that LANA-NBs are partially sensitive to LLPS disrupting 1,6-HD, but less sensitive to 2,5-HD in a time-frame similar to coilin-associated Cajal bodies.

### DAXX structures are resistant to LLPS disrupting agents

**Fig 2:**
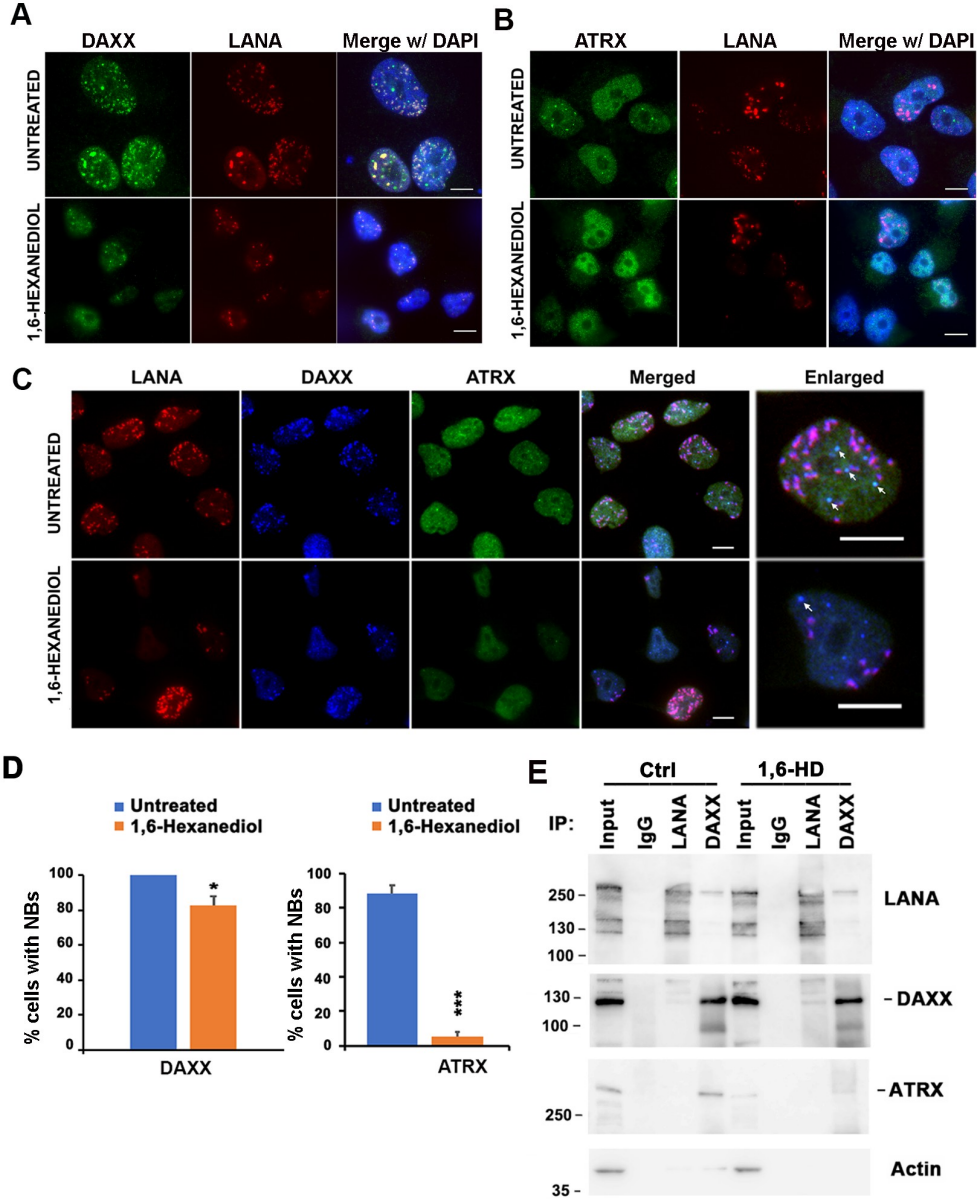
LLPS disrupting agent effects on DAXX and ATRX foci. **A**. iSLK RFP-LANA cells untreated or treated for 10 min with 3.5% 1,6-Hexanediol were imaged by IF for DAXX (green), RFP-LANA (red) or DAPI (blue). Scale bar = 10 μm. **B**. iSLK RFP-LANA cells untreated or treated for 10 min with 3.5% 1,6-Hexanediol were imaged by IF for ATRX (green), RFP-LANA (red) or DAPI (blue). Scale bar = 10 μm. **C**. Same as in panel A, except combined imaging with RFP-LANA (red), DAXX (blue), and ATRX (green). In enlarged images, arrows indicate colocalizations of ATRX and DAXX foci, but lacking LANA. Scale bar = 10 μm. **D**. Quantification of DAXX (left) and ATRX (right) foci in cells treated as described in panels A and B, respectively. Error bars are s.d., * = p value <0.05, *** = p value <0.001, two-tailed t-test, relative to untreated cells. **E**. IP-Western analysis of iSLK RFP-LANA cells treated with 3.5% 1,6-Hexanediol or DMSO control for 1 hr. Protein complexes were immunoprecipitated by antibodies to LANA, DAXX, or control IgG, and assayed by Western with antibodies specific for LANA, DAXX, ATRX, or Actin, as indicated. Molecular weight markers are indicated in KDa, and input represents 10% of cell lysis used for each IP.

We previously showed that LANA-NBs colocalize with DAXX, but not with ATRX or PML [18]. We therefore assayed the effects of LLPS disrupting agents on DAXX and ATRX and their relative colocalization with LANA-NBs (Fig 2 and S2 Fig). Using the same iSLK RFP-LANA cells and treatment conditions that were found to disrupt coilin and partially disrupt LANA foci (Fig 1 and S1 Fig), we found that DAXX NBs that colocalize with LANA-NBs were mostly resistant, while ATRX foci that do not colocalize with LANA were highly sensitive to 1,6-HD (Fig 2A–2D). We observed similar resistance to LLPS disrupting agents by DAXX in BCBL1 cells (S2 Fig). We also found that PML-NBs, as monitored by IF to PML, were highly resistant to LLPS disrupting agents (S2C and S2D Fig). In contrast, we found that ATRX NBs were reduced from 88% in untreated to ~5% in 6-HD treated iSLK RFP-LANA cells (Fig 2B–2D). These findings indicate that ATRX-NB are significantly disrupted, while DAXX-associated LANA-NBs and PML-NBs remain stable after treatment with LLPS disrupting agents. DAXX is known to co-immunoprecipitate (IP) with both LANA and ATRX. We therefore tested whether treatment with 1,6-HD altered the ability of LANA, DAXX or ATRX to coIP (Fig 2E). LANA co-IP with DAXX was detected in extracts from untreated, as well from 1,6-HD treated iSLK RFP-LANA cells. ATRX interaction with DAXX was readily detected in untreated iSLK RFP-LANA cells, but could not be detected in 1,6-HD treated iSLK RFP-LANA cells (Fig 2E). These findings indicate that DAXX interaction with LANA is not sensitive to LLPS, while both ATRX foci and interactions with DAXX are sensitive to LLPS disrupting agents.

### LLPS disruption does not induce KSHV lytic or inhibit LANA binding to KSHV genome

**Fig 3:**
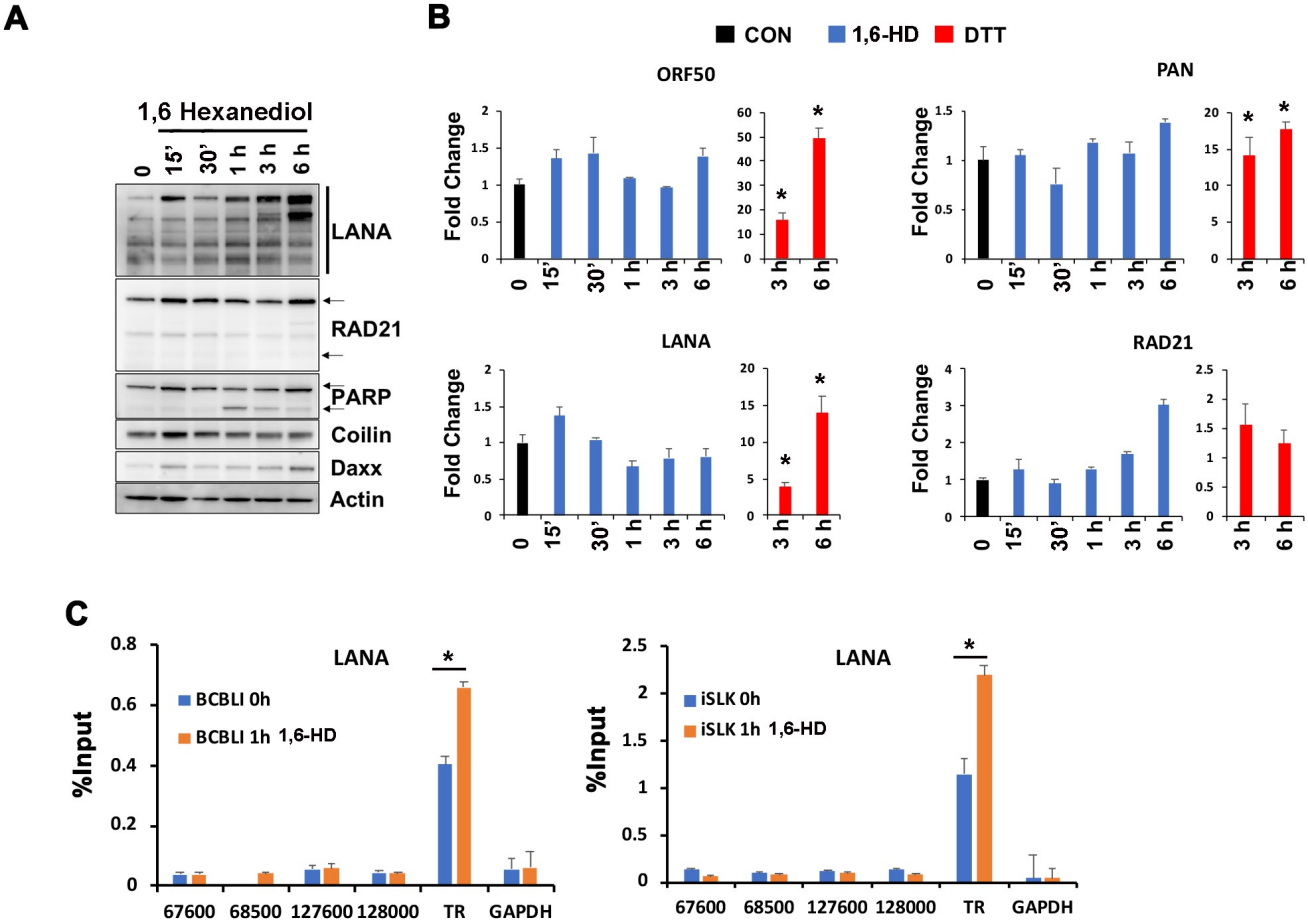
LLPS disrupting agent effects on KSHV transcription and LANA binding. **A**. Immunoblotting of LANA, RAD21, PARP1, Coilin, DAXX and actin in BCBL1 cells exposed to 3.5% 1,6-Hexanediol for the indicated times. **B**. RT-qPCR for lytic transcripts ORF50, PAN and latent transcript LANA relative to cellular actin in BCBL1 cells treated as in panel A. The data are expressed as fold change of the treated versus untreated cells. **C**. BCBL1 and iSLK RFP-LANA cells treated for 1 hr with 3.5% 1,6-Hexanediol were assayed by ChIP for LANA or IgG. ChIP-qPCR primer positions indicated on x-axis are relative to KSHV genome. GAPDH primers were used as internal control. * p value < 0.05, two-tailed t-test.

Previous studies have shown that LANA oligomerization is important for transcriptional silencing of viral genes [18]. We therefore tested the effects of LLPS disrupting agents on KSHV transcription using RT-PCR at various times after drug treatment. We found that 1,6-HD had a minor stabilizing effect on LANA protein and induced modest PARP1 cleavage after 1 hr of treatment as measured by Western blot (Fig 3A). In contrast,1,6-HD had no effects on coilin, RAD21, or DAXX protein levels (Fig 3A). RT-qPCR showed no significant increase in LANA or other KSHV transcripts at any time point up to 6 hrs post treatment (Fig 3B). Previous studies found that lytic transcription can be induced as early as 30 min after treatment with ER-stress inducing agents, such as DTT [18]. Consistently, we found that DTT treatment for 3 or 6 hrs led to >10 fold increase in lytic transcription for ORF50 and PAN RNA as demonstrated by RT-qPCR (Fig 3B). We also assayed whether 1,6-HD affected LANA binding to KSHV TR *in vivo* by ChIP assay (Fig 3C). Consistent with the increase in LANA protein expression, we found a small (~1.75 fold) increase in LANA binding to TR after 1,6-HD treatment in both iSLK RFP-LANA and BCBL1 cells. These findings indicate that 1,6-HD does not lead to lytic transcript reactivation, nor the loss of LANA binding to KSHV genomes, despite the loss of LANA NB morphology in 50% of the cells.

### LLPS disruption alters KSHV genome conformation

**Fig 4:**
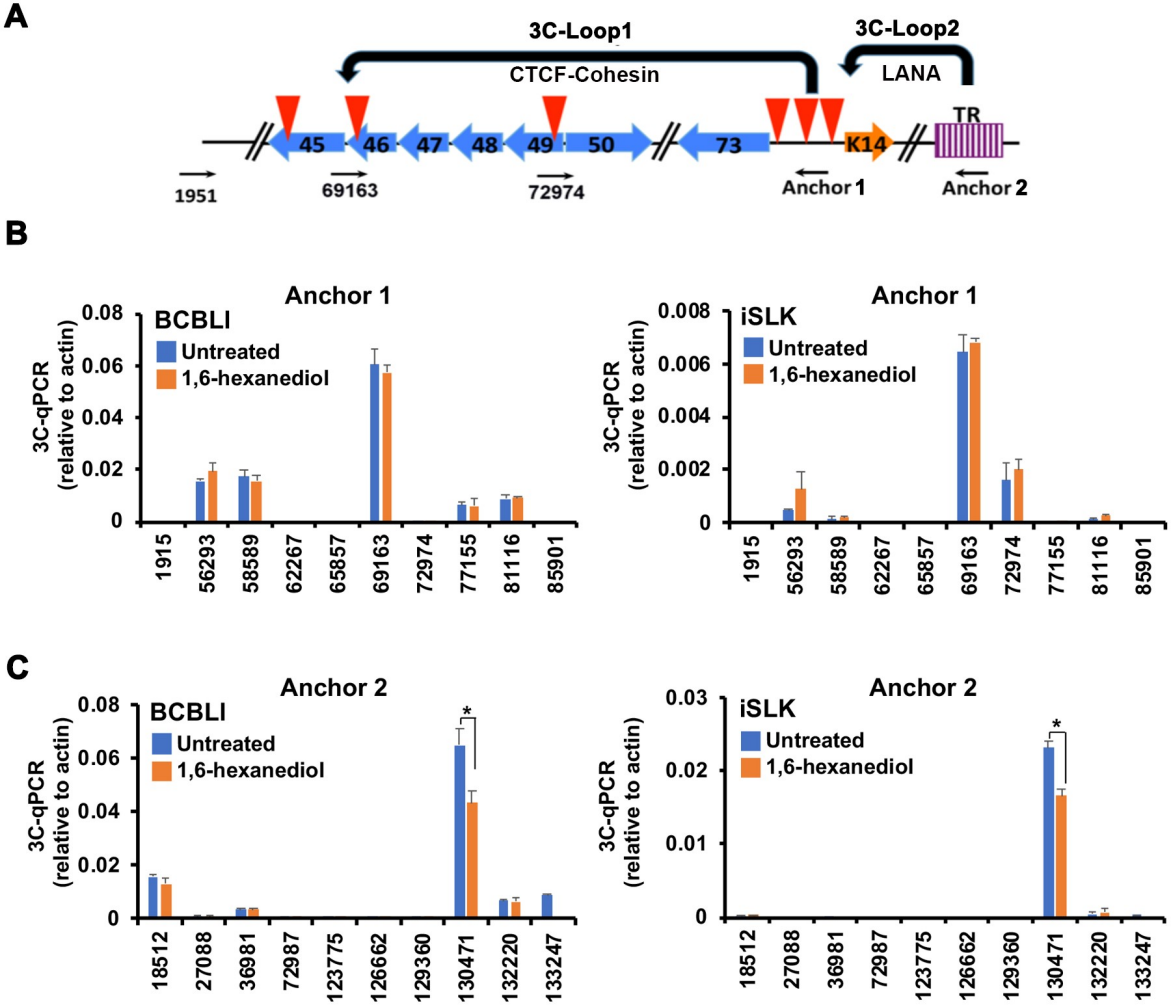
LLPS disrupting agent effects on KSHV chromosome conformation. **A**. Schematic of KSHV regulatory regions and primers used for 3C experiment with anchors for loop 1 (LANAp) or loop 2 (TR). Red arrowheads indicate previously mapped CTCF binding sites. **B**. BCBL1 and iSLK RFP-LANA cell lines treated for 1 hr with 3.5% 1,6-Hexanediol were assayed by 3C using anchor primer (Anchor 1) at latency control region (128,264) and acceptor primers at positions indicated on x-axis. 3C-qPCR relative to actin control is reported. **C**. BCBL1 and iSLK RFP-LANA cell lines treated as in panel B were assayed by 3C using anchor primer (Anchor 2) near TR (position 133872) and acceptor primers at positions indicated on x-axis. 3C-qPCR relative to actin control is reported. * p value < 0.05, two-tailed t-test.

Previous studies revealed that KSHV forms at least two distinct DNA-DNA loops as measured by chromosome conformation capture (3C) [18]. Loop1 occurs between the latent (LANAp) and lytic (ORF50p) control regions [38]. Loop2 is LANA-dependent loop between TR and LANAp region [18]. We therefore tested whether treatment with LLPS disrupting agents affected either of these 3C loops in both BCLB1 and iSLK cells (Fig 4A). We found that 1,6-HD led to a 30% decrease in Loop 2, but had no effect on Loop 1 in both latency models (Fig 4B and 4C). This suggests that the LANA-dependent 3C loop mediating interaction between TR and LANAp is regulated by LLPS dynamics, while the CTCF-cohesin interactions forming loop1 are not sensitive to LLPS disrupting agents.

### LANA-NBs form ring-like structures associated with KSHV DNA during lytic reactivation

**Fig 5:**
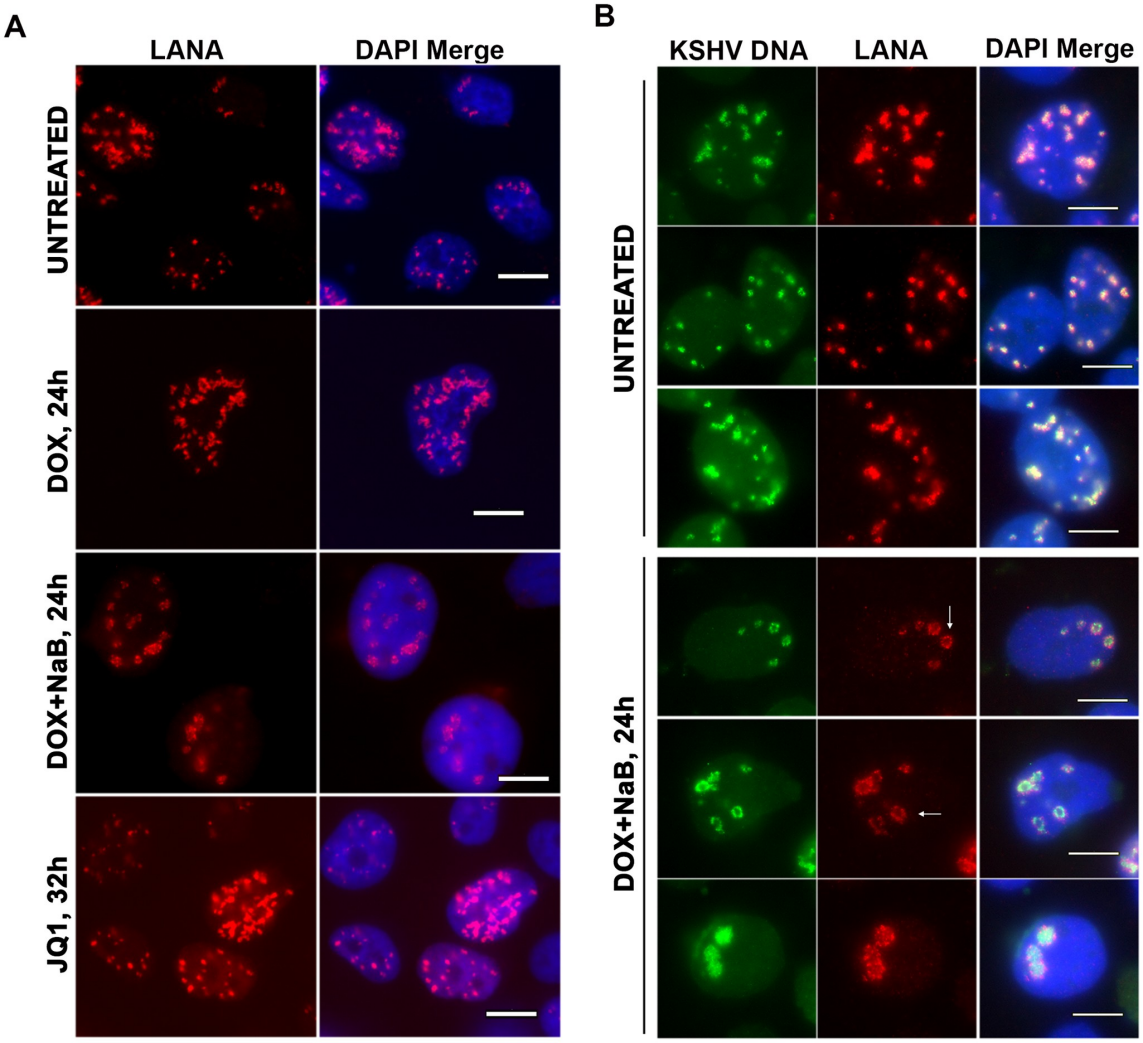
Dynamic changes in LANA-NB morphology after lytic induction. **A**. iSLK RFP-LANA cells untreated or induced by Dox or Dox+NaB for 24 h, or JQ1for 32 h were assayed by IF for RFP-LANA (red) and DAPI (blue). Scale bar = 10 μm. **B**. KSHV DNA FISH (green) combined with RFP-LANA IF (red) and DAPI (blue) in iSLK RFP-LANA cells untreated (top) or induced with Dox+NaB for 24h (lower panels). Scale bar = 10 μm. White arrows indicate examples of LANA ring-like structures.

LANA-NBs adopt several different nuclear morphologies in latent infection [18]. We next asked whether LANA-NBs undergo any morphological change during lytic reactivation from latency. We induced lytic cycle in iSLK RFP-LANA cells using either doxycycline (Dox) induced RTA, Dox-induced RTA + sodium butyrate (NaB), or JQ1, each of which has been shown to induce lytic cycle to a various extents [39]. We found that each of the lytic inducing agents increased the number, size, and fluorescence intensity of LANA-NBs per cell (Fig 5A). We also observed several distinctive morphologies, including the transformation of LANA-NBs into larger ring-like structures (Fig 5B). These ring-like structures were most prominent in Dox + NaB suggesting they correspond to more robust lytic reactivation. We next assayed LANA-NBs in combination with KSHV DNA FISH to determine the spatial relationship between LANA NBs and viral genomes during latency and reactivation (Fig 5B). We found that KSHV DNA colocalized with LANA NBs during latency and after lytic reactivation. In some cases, KSHV DNA colocalized with LANA along the circumference of the ring-like structures during lytic reactivation, while in other instances KSHV DNA appeared to increase within the center of these LANA-rings (Fig 5B).

### Spatial reorganization of KSHV DNA with DAXX and LANA-NBs during lytic reactivation

**Fig 6:**
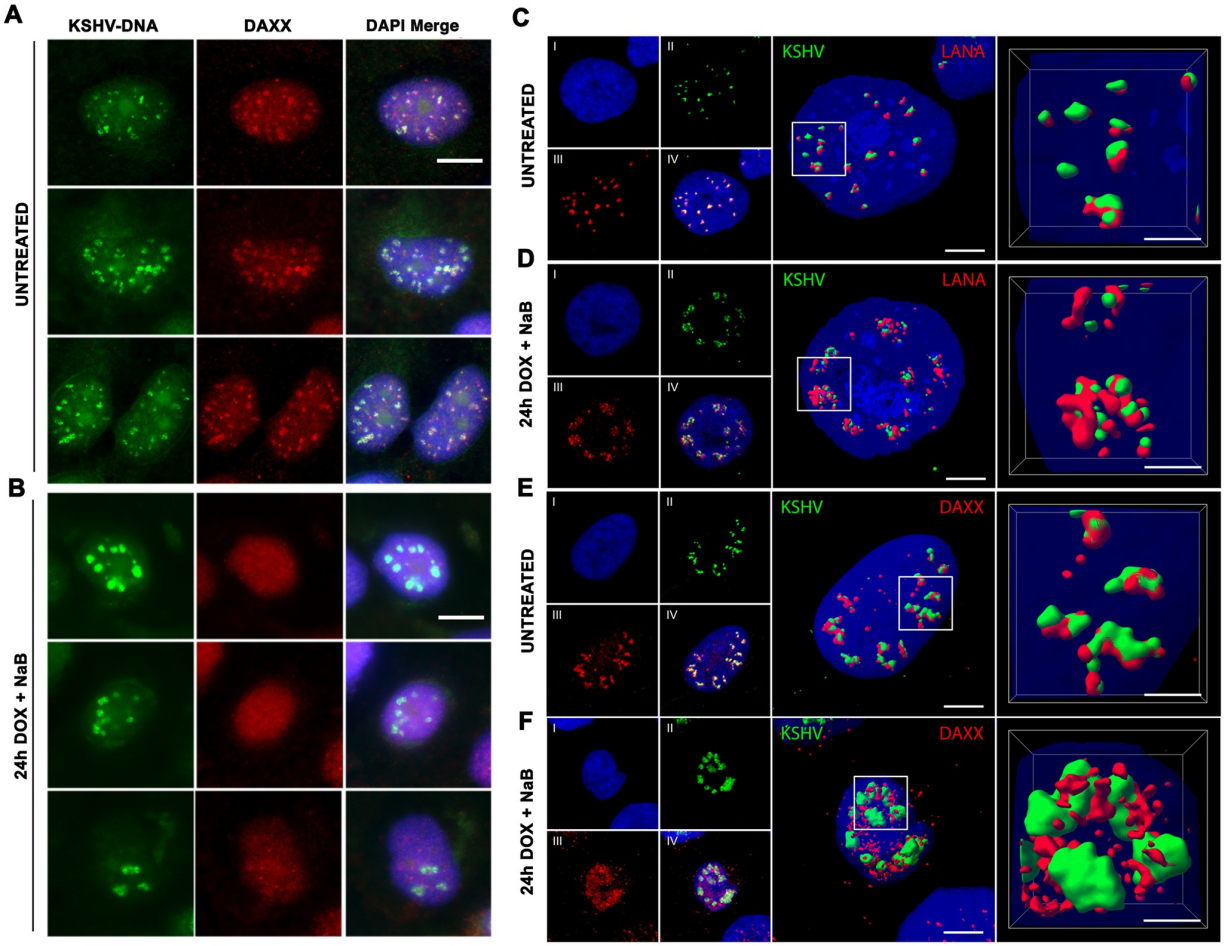
Redistribution of DAXX from LANA-NB during lytic induction. **A-B**. iSLK RFP-LANA cells untreated (A) or induced by Dox+NaB for 24 h (B) were imaged with KSHV-DNA FISH (green) or DAXX IF (red) and DAPI (blue). Scale bar = 10 μm. **C-D**. Confocal images and 3D reconstructions for iSLK RFP-LANA cells untreated (C) or induced by Dox+NaB (D) for 24h were imaged with KSHV DNA FISH (green), LANA IF (red) and DAPI (blue). Scale bar = 5 μm. **E-F**. Confocal images and 3D reconstructions of iSLK RFP-LANA cells untreated (E) or induced by Dox+NaB (F) for 24h were imaged with KSHV DNA FISH (green), DAXX IF (red) and DAPI (blue). Scale bar = 5 μm.

To investigate the relationship between DAXX and LANA-NB remodeling during lytic reactivation, we next assayed KSHV-DNA FISH in combination with DAXX-IF (Fig 6). In latently infected cells, we observed strong colocalization of DAXX with KSHV DNA (Fig 6A). This is consistent with previous studies showing strong colocalization of DAXX with LANA-NBs. However, during lytic reactivation, KSHV DNA associated with large ring-like nuclear body morphology, while DAXX was dispersed throughout the nucleus (Fig 6B). These findings suggest that DAXX is evicted from LANA-NBs and dissociated from KSHV DNA during reactivation.

We next used high-resolution confocal microscopy and 3D reconstruction to better analyze the spatial relationship between KSHV DNA and LANA or KSHV DNA and DAXX during latency and lytic reactivation (Fig 6C–6F and S1–S8 Movies). As expected, LANA colocalizes with KSHV DNA in latently infected cells, with most images indicating that LANA sits on compact surfaces of KSHV DNA (Fig 6C). This spatial relationship is altered during reactivation where both larger and more intense bodies of LANA and DNA appear to be interweaving, rather than completely overlapping (Fig 6D). DAXX also colocalizes strongly with KSHV DNA during latency, but appears to be extended over larger portions of the KSHV DNA surface than was observed for LANA (Fig 6E). In contrast, during lytic reactivation DAXX appears excluded from the larger KSHV DNA nuclear bodies and ring-like structures (Fig 6F). These findings confirm that KSHV reactivation leads to a change in the spatial re-organization of LANA and KSHV genomic DNA, and the redistribution of DAXX from LANA-NBs during lytic reactivation.

**Fig 7:**
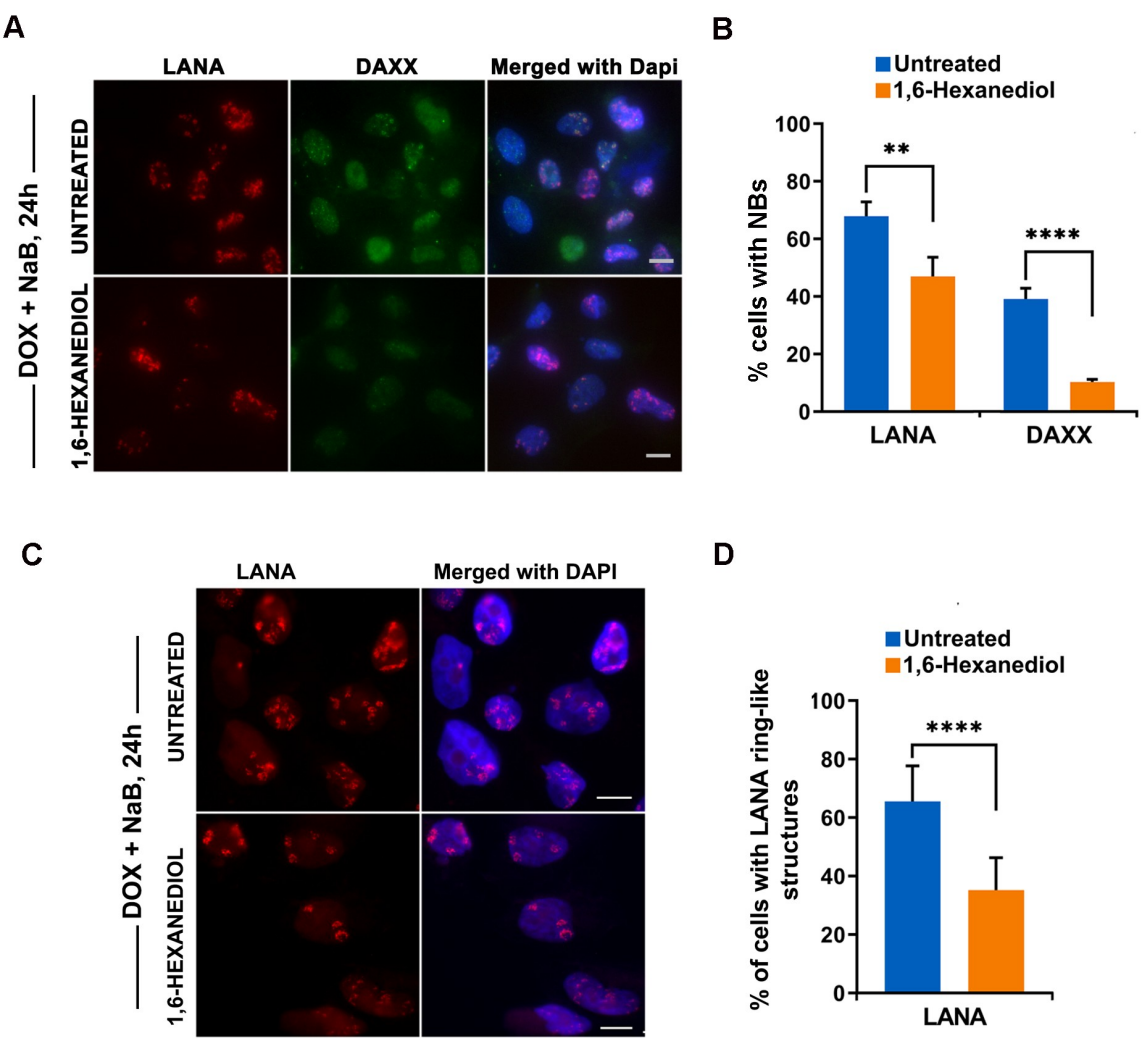
Sensitivity of lytic induced LANA ring-like structures to LLPS. **A**. iSLK cells were induced with Dox+NaB for 24h and then untreated or treated with 3.5% 1,6-HD for 10 min followed by IF for RFP-LANA (red), DAXX (green) or DAPI (blue). Scale bar = 10 μm. **B**. Quantitation of DAXX or LANA foci for experiments represented by panel A. N = 200 cells, **** p < .001, ** p < .01 two-tailed student t-test. **C**. Same as in panel A, but IF for RFP-LANA (red) and DAPI (blue). Scale bar = 10 μm. **D**. Quantification of IF images represented by panel A for % of cells with LANA ring-like structures. N = 200 cells, ****p < .001, two-tailed student t-test.

We next tested whether LANA ring-like structure colocalize with KSHV lytic cycle replication proteins, such as K8 or ORF45 (S3 and S4 Figs). While K8 is a b-zip protein implicated in DNA replication, it is not known if it colocalizes with lytic replication compartments. After 24 h induction with Dox + NaB, we found most of the K8 and ORF45 as pan-nuclear, and very few cells with punctate foci. Using K8 or ORF45 as markers for lytic cycle induction, we found that ~70% of the K8 or ORF45 lytic cells had LANA ring-like structures (S3 Fig) indicating that most, but not all lytic cells form these LANA superstructures. We observed rare cells with K8 punctate foci that partly colocalized with LANA ring-like structures (S4 Fig), suggesting that colocalization may occur at earlier stages of lytic cycle replication. We also tested whether LANA ring-like structures were sensitive to 1,6-HD using treatment conditions identical to those used for latent cycle LANA-NBs. We analyzed the results in two ways. We first quantified the % of cells with DAXX or LANA-NBs (Fig 7A and 7B). This revealed that 1,6-HD reduced DAXX foci by ~5 fold, while having less than a 2-fold reduction of LANA-NBs (Fig 7B). We also counted more specifically the LANA ring-like structures. These structures were found to be reduced by ~3 fold after 1,6-HD treatment (Fig 7C and 7D). Taken together, these findings suggest that both LANA-NBs and lytic induced LANA ring-like superstructures are partially dependent on LLPS.

**Fig 8:**
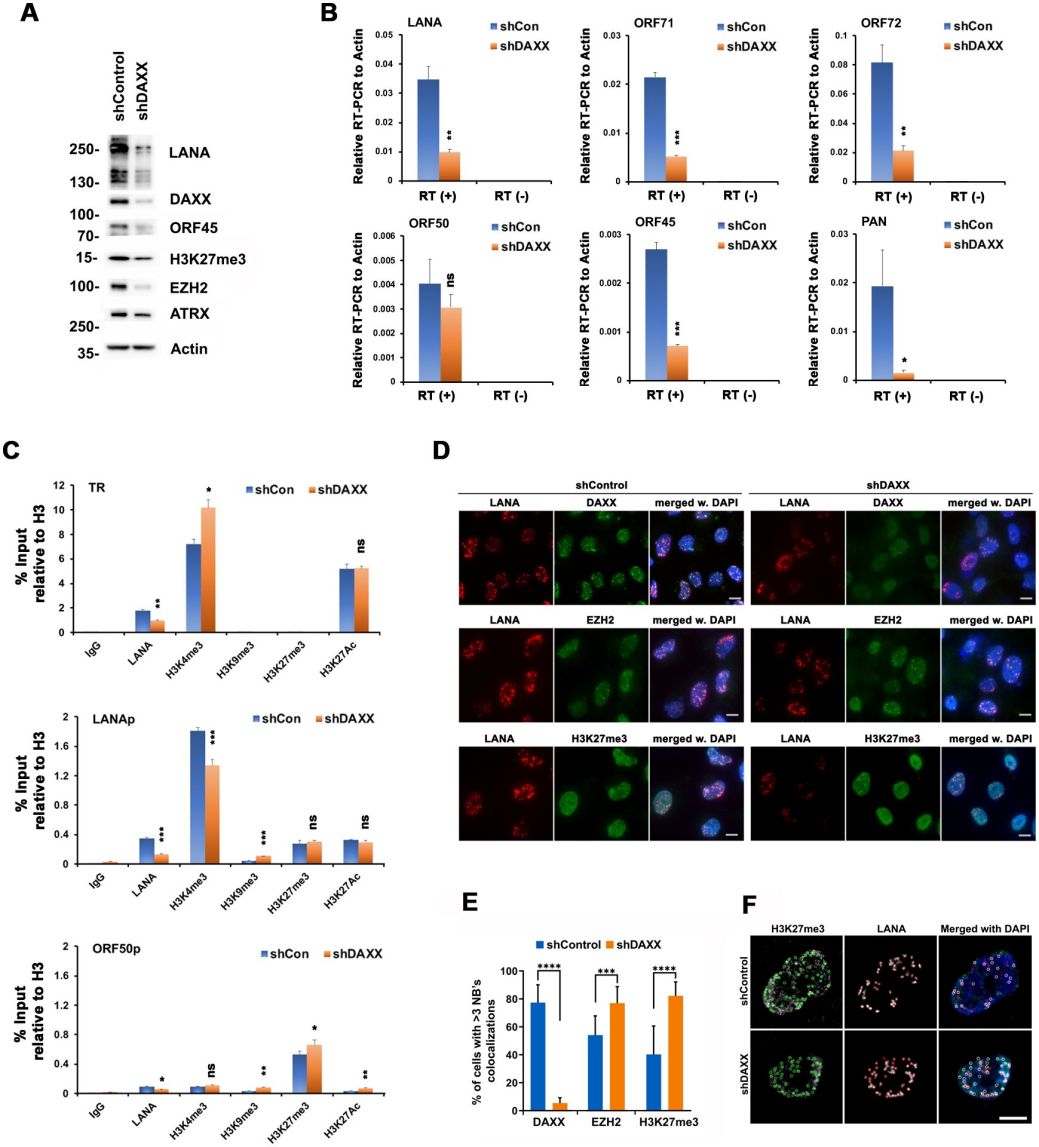
Effects of shDAXX depletion on KSHV transcripts, epigenetics, and LANA-NBs. **A**. Western blot analysis of iSLK RFP-LANA cells infected with lentiviruses encoding DAXX or control shRNAs for 6 days with antibodies specific for LANA, DAXX, ORF45, H3K27me3, EZH2, ATRX, or Actin, as indicated. Molecular weight markers are indicated in KDa. **B**. iSLK RFP-LANA cells treated as in panel A were assayed by RT-qPCR at Day 6 for KSHV latency-associated genes LANA, ORF71, ORF73 or lytic-associated genes ORF50, ORF45, PAN. The bar graph represents means ± s.d. from three independent DAXX depletion experiments. * p value <0.05, ** p <0.01, *** p < 0.005, ns not significant using two-tailed t-test. **C**. iSLK RFP-LANA cells infected with shCon or shDAXX for 6 days were assayed by ChIP using antibodies against IgG, LANA, histone H3K4me3, H3K9me3, H3K27me3, and H3K27Ac, and normalized to total H3 ChIP signal for each loci, as indicated. ChIPed DNA was examined by qPCR using primers for KSHV TR (top panel), LANA promoter (middle panel), or ORF50 promoter (bottom panel). Bar graph represents the average value of percentage of input relative to H3 for each ChIP from three independent PCR reactions (mean ± s.d.). * p value <0.05, ** p <0.01, *** p < 0.005, ns not significant using two-tailed t-test. **D**. iSLK RFP-LANA cells infected with shCon or shDAXX for 6 days were assayed by IF for RFP-LANA (red) and either DAXX (top), EZH2 (middle), or H3K27me3 (lower) in green, and merge with DAPI (blue). Scale bar = 10 μm. **E**. Quantification of IF images represented by panel D for % of cells with >5 colocalizations of RFP-LANA foci with either DAXX, EZH2, or H3K27me3 foci. N = 200 cells, ***p <0.005, ****p < .001, two-tailed student t-test. **F**. Example of computational analysis for quantifying colocalization of LANA and H3K27me3 foci. The colored circular outlines indicate the number of H3K27me3 (green) and RFP-LANA (red) foci. Bar scale = 10μm.

The function of DAXX in LANA-NB formation and KSHV regulation is not clear. Others have found that DAXX depletion has little effect on KSHV gene expression or lytic replication in BCBL1 cells [40]. We found that partial depletion of DAXX in KSHV iSLK cells led to a decrease in KSHV LANA and ORF45 protein (Fig 8A), and corresponding decreases in viral gene transcripts (Fig 8B). We also observed a reduction in EZH2 protein (Fig 8A). The effect of DAXX depletion on the viral chromatin was assayed by ChIP assay (Fig 8C). DAXX depletion reduced LANA binding and altered histone modifications at several key viral control regions including TR, LANAp and ORF50p (Fig 8C). Consistent with the decrease in viral transcription, we observed a loss of H3K4me3 at the LANAp and an increase of H3K9me3 and H3K27me3 at LANAp and ORF50p, respectively (Fig 8C). ChIP assay also revealed an increase in H3K4me3 at the TR. We next examined the effects of DAXX shRNA depletion by IF (Fig 8D). DAXX depletion had no obvious effect on LANA-NBs morphology, although average fluorescence intensity of LANA-NBs was reduced. DAXX-depletion also increased the colocalization of EZH2 and H3K27me3 foci with LANA-NBs (Fig 8D–8F). Taken together, these findings indicate that DAXX associates with LANA-NBs during latency and contributes to epigenetic programming of the latent KSHV genome.

## Discussion

Macromolecular self-assembly and membrane-less compartments provide vital functional utility for many subcellular biological processes [41–43]. Viruses are known to induce formation of a diverse intracellular structures and compartments required to complete different aspects of their life cycle within the host cell [44]. Compartments for viral DNA replication [45,46], capsid assembly [47], RNA processing [48], and protein quality control [49] have been described for numerous viruses. Here, we further characterize the dynamic properties of the LANA-NBs that have been implicated in episome maintenance during KSHV latent infection. While we have previously shown that these LANA-NBs remain organized during latent cycle cell division and undergo orchestrated distribution to daughter cells during mitotic cell division [18], we now show that these structures are partly dependent on phase separation dynamics and undergo morphological transition during the lytic reactivation cycle. We show that LLPS disrupting solvents reduce LANA-NB formation as well as LANA-dependent chromosome conformation of the KSHV genome. Finally, we show that DAXX, but not ATRX, associates with LANA-NBs during latency, and is dispersed from LANA-NBs during reactivation. Taken together, these findings indicate the LANA-NBs are dynamic super-molecular nuclear structures that undergo morphological transitions corresponding the functional state of the viral genome.

### LLPS contributes to LANA-NB formation

**Fig 9:**
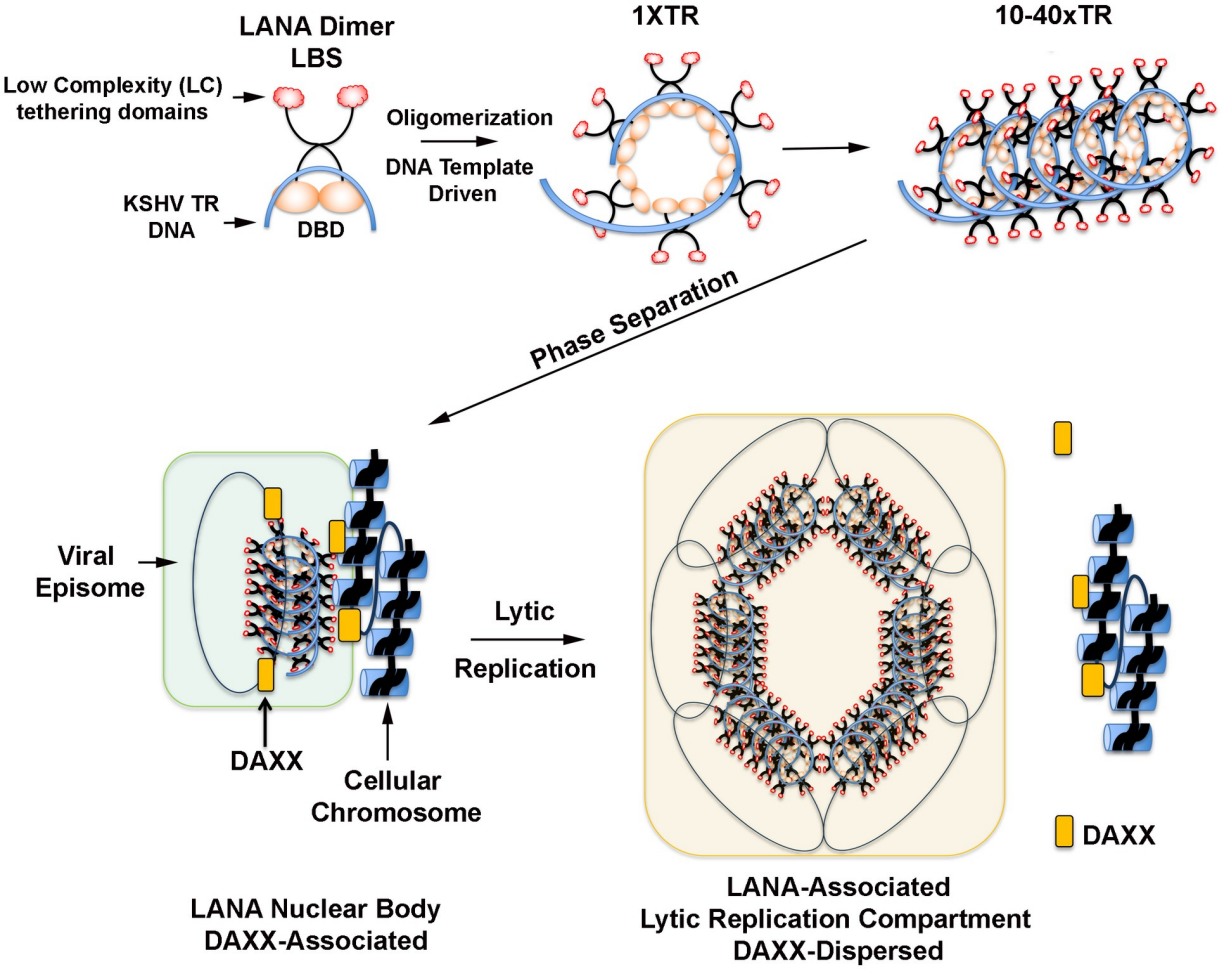
Model of LLPS-driven LANA-NB formation. Dynamic LANA-NB structures form through the combined actions of the structured DNA binding domain (DBD), KSHV terminal repeat DNA templated oligomerization, and the low complexity, multivalent N-terminal domain interactions driving LLPS.

LLPS involves the condensation of a critical mass of multi-valent components [28,50]. In many cases, these structures are dependent or facilitated by nucleic acid templates, including RNA and DNA [31,51]. LANA-NBs are known to form only when LANA is expressed at sufficient concentration in combination with KSHV terminal repeat DNA [11,52]. The cooperative binding of LANA to 3 adjacent sites in each of the 10–40 tandem copies of the TR is likely to provide sufficient local concentration of LANA to drive phase change reactions. Proteins that form LLPS typically have low-complexity (LC) domains that are inherently unstructured but can adapt conformations that enable a variety of different macromolecular shapes to accommodate diverse ligand interactions. LANA binds to the viral template DNA with its highly structured C-terminal DNA binding domain that is inherently prone to form higher-ordered oligomeric structures, including pentamers, decamers and continuous helical spirals [10,18,20]. In addition to its DNA binding domain, LANA also has low complexity (LC) domains involved in various interactions of functional significance. The N-terminal basic domain can bind directly to an acidic patch in the nucleosome formed by histones H2A and H2B [21]. The proline rich domain (aa194-273) contains sites for protein destabilization, SUMO-2 interaction and KAP1 repressor binding [53,54]. The acidic rich domain of LANA can interact directly with DAXX [55]. The acidic domain of LANA has been implicated in many other interactions important for transcription regulation, including binding to CBP [56]. We propose that LANA-NBs form through a combination of rigid and flexible structures, including a KSHV TR DNA template driven oligomerization of the rigid DBD and an LLPS driven multivalency of the N-terminal LC domains. This combination provides structural flexibility required for viral transcription and replication in both latent and lytic stages of the viral life cycle (Fig 9).

One major limitation of these studies, along with many others in LLPS investigation, is the reliance on a limited number of organic molecules like 1,6-HD to evaluate LLPS. These organic molecules can have pleiotropic effects on other cell structures and processes. However, the selective effects of 1,6-HD relative to 2,5-HD supports the model that LLPS contributes to the formation and dynamic properties of LANA-NBs in both latent and lytic cycle structures.

### KSHV replication compartments

We found that LANA-NBs undergo morphological change during latent and lytic cycles and remain closely associated with viral DNA. During lytic reactivation, LANA-NBs formed large ring-like structures with LANA enriched along the perimeter and KSHV DNA localized mostly within the rings. Confocal imaging suggests that the lytic replication compartments are two-dimensional rings and not cross sections of globular spheres. KSHV replication compartments have been described in other studies [57–60]. Replication compartments similar to what we observed have been described and shown to colocalize with DNA damage repair factors associate with NHEJ, including DNA-PK, ATM, and MRN components [57]. KSHV RNAs, especially the non-coding RNA PAN, has been shown to localize to KSHV replication compartments, suggesting that RNA also contribute to the formation of these super-molecular structures [48]. KSHV lytic replication factors by themselves can self-assemble into subnuclear compartments that colocalized near PML-NBs, but these did not depend on LANA or viral DNA templates [61]. In contrast, we found that KSHV DNA and LANA did not colocalize with PML-NBs. Although DAXX is typically found associated with PML-NBs, it associates with LANA-NBs separate from PML during KSHV latency. This colocalization is disrupted during lytic reactivation, when LANA tended to form large ring-like structures that colocalized with expansion of KSHV DNA. We observed rare punctate colocalization of KSHV lytic replication protein K8 with LANA rings, suggesting that these structures may be distinct from classical replication compartments described for other viruses. LANA has also been reported to accumulated in cytoplasmic foci during lytic replication cycle [60], but our experimental system using RFP-LANA may only observe full length LANA in the nucleus. While LANA is well known for its role in KSHV latent cycle replication, our new findings suggest that LANA also contributes to the structural organization of KSHV lytic cycle DNA replication. We propose that LANA has a fundamental architectural role in both latent and early phase of the lytic replication cycles of KSHV.

### DAXX eviction from LANA-NBs and KSHV DNA during reactivation

KSHV-NBs colocalize with DAXX but not with PML, or other components of the PML-NBs [18]. We show here that ATRX is not associated with LANA-NBs, and that it behaves differently than DAXX in response to LLPS disrupting agents. DAXX is known to function in association with ATRX as a histone H3.3 chaperone [62], but is also known to interact with other proteins, including LANA [55]. Exclusion of DAXX from lytic replication compartments may reflect the block to histone loading on newly replicating viral DNA [36]. The interaction interfaces of LANA and DAXX are both low complexity domains suggesting they may utilize LLPS dynamic interactions. The function of DAXX in LANA-NB formation and KSHV regulation is not clear. While DAXX depletion led to changes in KSHV gene expression and histone modifications in iSLK cells, we and others have also found that DAXX depletion has only a small effect on KSHV gene expression or lytic replication in BCLB1 cells [40]. Furthermore, DAXX depletion has no obvious effect on LANA-NB morphology. The discrepancy of effects of DAXX depletion on KSHV gene expression and chromatin regulation in iSLK and BCBL1 cells remain elusive. DAXX can also interact with SUMO-modified proteins that may regulate protein trafficking and folding [63]. While an essential role of DAXX in LANA-NB formation is not known, it is likely that DAXX contributes to the LLPS dynamics of LANA and morphological change of LANA-NBs during the different stages of KSHV life cycle.

### Role of LLPS in viral assemblies

LLPS and biomolecular condensate forces are likely to drive formation of many biological structures, especially rapidly transitioning viral compartments [36]. Replication compartments for several viruses, including measles and VSV, are known to be LLPS dependent [64,65]. Super-enhancers induced by viral transcription factors, such as EBV EBNALP were found to be LLPS dependent [66]. Numerous viral-specific structures are also suspected of being LLPS dependent [26,36]. CMV assembly compartments [47], viral induced chaperone-enriched (VICE) domains that serve as nuclear protein quality control centers [49], AdV replication compartments undergo morphological changes during replication and viral maturation [44], and HSV replication compartments that can fuse to form larger superstructures [45,46], are all likely to involve LLPS. However, a recent study found that HSV replication compartments were mostly insensitive to LLPS disrupting agents [67]. We found that only a small percentage of LANA NBs are dispersed by LLPS disrupting agents, indicating that physical forces other than LLPS contribute to the formation and stability of LANA-NBs. LANA-NBs are highly stable during latency, and rather than disperse, are remodeled and repurposed into replication compartments during lytic reactivation. It is likely that LANA-NBs utilize combinations of interaction forces, including LLPS-dependent, to form malleable gel-like polymers [68]. Since LANA-NB are “templated” by repetitive DNA [33], they may have properties that distinguish them from lower complexity structures. In conclusion, LANA-NBs have dynamic structural properties involving phase separation condensates of low-complexity domain interactions in addition to stable solid-state components providing the necessary plasticity for genome conformational changes associated with diverse transcriptional responses and different modes of viral DNA replication. Such modular structures provide protection and adaptability necessary for viral persistence and transmission.

## Methods

### Cell culture

iSLK cells (gift from Don Ganem, Novartis) [69] were cultured in Dulbecco’s Modified Eagle’s medium (DMEM) supplemented with 10% fetal bovine serum (FBS, heat inactivated) and 1% penicillin-streptomycin (P/S) in presence of 1μg/ml puromycin and 250 μg/ml G418. iSLK stable cell lines carrying KSHV Bac16 expressing RFP-LANA were cultured in iSLK-growth medium with additional 1200 μg/ml Hygromycin B. BCBL1 cells [70], a stable cell line derived from KSHV^+^ pleural effusion lymphoma (gift from Yan Yuan, UPENN) were cultured in RPMI-1640 medium supplemented with 10% FBS (heat inactivated) and 1% P/S. LLPS disrupting agent 1,6-Hexanediol (SIGMA, 240117), 2,5-Hexanediol (SIGMA, H11904) or 1,5-Pentanediol (SIGMA, 76892) were dissolved in cells growth medium at concentrations and durations indicated. For induced lytic cycle in iSLK RFP-LANA cells were treated either with 1μg/mL doxycyclin (DOX), and/or with 1mM sodium butyrate (NaB), or with 4μM JQ1 (Sigma-Aldrich, SML1524) dissolved in the cells growth medium.

### Bacmids, plasmids and BAC16 iSLK-cell line

The KSHV bacmid BAC16 was kindly provided by Dr. Jae U. Jung and K. Brulois (University of Southern California) [71]. The RFP-LANA bacmid was created from KSHV BAC16 clone as described previously [18].

### Indirect immunofluorescence (IF) assay

For adherent cells (iSLK), 1x10^5^ (80% confluence) cells were plated on 12 mm diameter glass coverslips in 24-well plate. For suspension cells (BCBL1) cells were collected, washed with 1xPBS and plated on the 12 mm glass coverslips in amount 1x10^5^ using the Cytospin (SHANDON) at 1000 rpm for 5 minutes, on high acceleration. Cells mounted on slides were washed with 1XPBS and fixed for 15 min with 2% paraformaldehyde (Electron Microscopy Sciences) in 1XPBS, washed twice with 1XPBS and permeabilized with 0.3% TritonX-100 (Sigma) in PBS. All procedures were performed at room temperature. After washing with PBS, slides were incubated in blocking solution (0.2% fish gelatin, 0.5% BSA in 1XPBS) for 30 min. Primary antibodies was diluted in blocking solution and applied on cells for 1h followed with 1xPBS washing. Cells were further incubated with fluorescence-conjugated secondary antibodies in blocking solution for 1h, counterstained with DAPI and mounted in SlowFade Gold mounting solution (LifeTechnologies). Images were acquired with a Nikon 80i Upright Microscope (Nikon Instruments) at 60X or 100X lens using Nikon ImagePro Plus software (Media Cybernetics) and Adobe Photoshop CS6 for image capture and processing.

### Fluorescence in situ hybridization (FISH)

After fixation in 1% paraformaldehyde solution for 10 minutes and permeabilization in 0.3% TritonX-100 for 15 min cells were washed with 1xPBS, dehydrated with ethanol wash series and air dried. Cells were hybridized with KSHV BACMID probe (labeled with biotin (Invitrogen, Cat # 18247–015) and Oregon Green 488 (Invitrogen cat # A6374) in Hybridization Buffer B (Cytocell) overnight at 37°C. Slides were washed two times for 5 min each with Wash1 (50% Formamide, 10 mM Tris pH 7.5, 0.1% Tween 20, 0.1% BSA), three times for 5 min each with Wash 2 (50mM Tris pH 7.5, 150mM NaCl, 0.1% BSA, 0.1% Tween 20), and then stained with DAPI. Slides were dehydrated with ethanol wash series, air dried and mounted in SlowFade Gold mounting solution. Images were taken at 60X or 100X zoom using Nikon 80i Upright Microscope and processed with Adobe Photoshop CS6.

### Confocal microscopy and image processing

High resolution, confocal images of fixed cells were captured using a Leica TCS SP8 WLL scanning laser confocal microscope and Leica LAS-X software (Leica Microsystems, Inc., Buffalo Grove, IL). Image post-processing included importing into Huygens software for deconvolution (Scientific Volume Imaging, Laapersveld, Hilversum, The Netherlands) followed by 2D maximum projection or 3D reconstruction, iso-surface application and video rendition in LAS-X.

Fixed cell preparations were acquired using a 63X/1.40 oil objective, 6X zoom and a pinhole of 1 AU, with 11–15 z-steps through 3–3.5 um stacks, resulting in a voxel size of 60 x 60 x 299nm. Cells were labeled with DAPI (nuclei), Alexa488 (KSHV) and RFP (LANA or DAXX) and acquired with HyD detectors in sequence to maximize signal and minimize cross-talk. Foci and colocalizations were determined and quantified using Nikon NIS Elements AR software, version 5.02, using the Spot Detection Tool. Using the Nikon NIS-Elements AR software, version 5.02 with the Bright Spot Detection Tool, the size of the smaller single LANA dot = 0.79μm, the bigger LANA structure = 2.87μm. Images acquired with the Nikon Upright Microscope utilized the Image-Pro Plus 7.1 software with saturation and exposure time tools to limit signal saturation. ImageJ 1.49 processing program was used to count only NBs that were not overexposed.

Colocalization was determined and quantified with Nikon NIS-Elements AR software, version 5.02 using the Bright Spot Detection Tool. For each detected nuclear body, a 1μm circular binary outline is stored. For the detected objects, the growing feature adjusted to match the object’s shape. The same procedure was used for either LANA and DAXX, or LANA and EZH2 NBs colocalizations.

For 3D reconstructions, original confocal files were captured using a Leica TCS SP8 WLL scanning laser confocal microscope (Leica Microsystems, Inc., Buffalo Grove, Ill.). Extended focus Z-stacks of each cell were acquired according to optimized settings based on Nyquist criteria and the resulting files were processed using Huygens deconvolution software (Scientific Volume Imaging, B.V., Hilversum, Netherlands). 3D reconstructions and video animations were then created using the Leica LAS-X 3D software module.

### Western blot analysis

Cells were collected in RIPA buffer (50mM Tris-HCl pH 8.0, 150mM NaCl, 1% NP-40, 0.5% Sodium deoxycholate, 0.1% SDS, 1mM EDTA). Equal amounts of protein extracts were resolved in 8–16% Tris-Glycine gels and then transferred onto PVDF membrane followed with specific antibodies application. Antibody signal was detected using Luminata HRP detection reagent (Millipore) and Luminescent Imager 680 (Amersham Biosciences).

### Antibodies

The following antibodies were used for immunofluorescence, Co-IP, and Western blotting studies: rat polyclonal anti-HHV8 or anti-LANA [LN53] (Abcam, ab4103), rabbit anti-Coilin (Cell Signaling technology,14168S), rabbit anti-DAXX (Sigma, D7810), rabbit anti-PML (Bethyl, A301167A), mouse anti-ORF50 (provided by Erle Robertson, UPENN), mouse anti-ORF45 (provided by Yan Yuan, UPENN), mouse anti-EBV (BIO-RAD, 4260–0906), anti-RAD21 (Abcam ab992), rabbit polyclonal anti-PARP1 (Enzo ALX-210-302-R100), rabbit EZH2 (Cell Signaling, 4905), rabbit H3K27me3 (Active Motif, 39155), rabbit IgG (Santa Cruz Biotechnology), AlexaFluor594 or AlexaFluor488 (Invitrogen), and mouse anti-Actin-HRP (Sigma, A23852).

### RT-qPCR

Total RNA was isolated from BCBL1 cells treated with either 3.5% 1, 6-HD or DTT at indicated times using Trizol reagents (Life Technologies) as manufacturer’s instructions. For RT in shDAXX depleted cells, total RNA was isolated from KSHV iSLK cells infected with lentiviruses encoding shDAXX or shControl at day 6 post-infection and puromycin selection. RT reaction was performed by using Superscript IV Reverse Transcriptase (Invitrogen) as specified by the manufacturer. qPCR was performed using a SYBR green probe with ABI Prism 7900 Sequence Detection System (Applied Biosystems). Relative RT-PCR was determined using ΔΔCT methods relative to control gene *Actin* or *Gapdh*, as indicated. Primers used in RT-qPCR are listed in [18].

### Co-Immunoprecipitation (Co-IP)

Co-IP assays were performed essentially as described previously [72]. About 5 x 10^6^ KSHV iSLK or BCBL1 cells mock-treated or treated with 3.5% 1, 6-HD for 1 hr were lysed in 1ml cold lysis buffer (20 mM Tris, pH 7.5, 137 mM KCl, 1 mM EDTA, 1.5 mM MgCl_2_, 10% glycerol, 1% Triton X-100) fresh supplemented 1 mM DTT and protease inhibitor cocktails (Sigma) for 30 mins. Cell lysates were collected by centrifugation at 14000 rpm for 10 mins at 4^**o**^ C, and used for IP by antibodies to LANA, DAXX, and control IgG at 4^**o**^ C overnight with rotation. The immunoprecipitates were collected by sepharose A + G beads and washed three times 10 mins each with BC200 (20 mM Tris, pH 7.5, 0.2 mM EDTA, 10% glycerol, 200 mM KCl, 10 mM β-mercaptoethanol, 1 x protease inhibitor cocktails) and once for 20 mins with BC100 at 4^**o**^ C. The immune-complex was eluted from beads with 2x Laemmli buffer and subject to Western blot analysis.

### Chromatin Immunoprecipitation (ChIP) assays

ChIP assays were performed as described previously with minor modifications [73]. Briefly, KSHV iSLK or BCBL1 cells were mock treated or treated with 3.5% 1, 6-HD for 1 hr. The cells (~ 1 x10^7^) were crosslinked in 1% formaldehyde for 15 min with vigorous shaking at room temperature, quenched in 0.125 M glycine for 5 min, and washed two times with PBS prior to ChIP lysis. For ChIP in DAXX depletion, iSLK cells were infected with lentiviruses encoding shDAXX or shControl. At 48 hrs post-infection, cells were expanded and cultured under puromycin (1 μg/ml) selection for 4 days. At day 6 post-infection, the selected cells were collected and subject to ChIP analysis. The antibodies used in ChIP assays include rat polyclonal anti-HHV8 or anti-LANA [LN53] (Abcam, ab4103), rabbit anti-H3K4me3 (EMD Millipore, 07–473), rabbit anti-H3K9me3 (Diagenode, C15410056), rabbit anti-H3K27me3 (Active Motif, 39155), rabbit anti-H3K27Ac (Abcam, ab4729), rabbit anti-H3 (EMD Millipore, 07–690), and rabbit IgG (Santa Cruz Biotechnology).

### Chromatin conformation capture (3C)

ChIP assays were performed as described previously [73]. 3C experiments were carried out as previously described [18] using for 3C-loop1 primers listed in [38] and for 3C-loop2 primers listed in [18].

## Supporting information

S1 Fig Dose titration and time course for treatment and recovery with 1,6-Hexanediol. **A**. BCBL1 cells were untreated or treated with 1.0, 2.0, or 3.0% 1,6-HD for 15 min and imaged by IF for Coilin(green) or LANA (red) or merged with DAPI (blue). Scale bar = 10 μm. **B**. BCBL1 cells were treated with 3.5% 1,6-Hexanediol for either 10, 20, or 30 min and imaged by IF as in panel A. Scale bar = 10 μm. **C**. BCBL1 cells were treated for 30 min with 3.5% 1,6 Hexanediol (top panel) followed by recovery in fresh media for 10, 20, or 30 min and imaged by IF as in panel A. Scale bar = 10 μm. **D**. iSLK cells were treated with 3.5% 1,6-HD for 10, 20, or 30 min and imaged by IF as in panel A. Scale bar = 10 μm.
(TIF)

S2 Fig PML-NBs are resistant to LLPS disruptors. **A**. iSLK RFP-LANA cells were either untreated or treated with 3.5% 1,6-HD or 1,5-PD for 10 min and then assayed by IF for LANA (red) or DAXX (green) or merged with DAPI (blue). **B**. BCBL1 cells were treated same as in panel A, except IF with LANA (red), and DAXX (green). **C**. iSLK RFP-LANA cells treated as in panel A, but IF with PML (green) and merged with DAPI (blue). **D**. BCBL1 cells treated as in panel C, except IF with LANA (red) and PML (green). Scale bar = 10μm.
(TIF)

S3 Fig Frequency of LANA ring-like structures in cells expressing lytic proteins K8 and ORF45. **A**. iSLK RFP-LANA cells were either untreated or induced with Dox+NaB for 24 h and imaged by IF for K8 (green), RFP-LANA (red), and DAPI (blue). Scale bar = 10μM. **B**. Quantification of cell images represented in panel A, for percent of cells with LANA ring-like structures for all LANA positive cells (blue) or K8 positive cells (yellow). The bar graphs represent mean± s.d., p value not significant (ns), two tailed t-test, relative to total LANA rings. **C**. Same as in panel A, except with ORF45 (green). **D**. Quantification for panel C, as described in B.
(TIF)

S4 Fig Rare colocalization of K8 with LANA rings. iSLK RFP-LANA cells were induced with DOX+NaB for 24 h and imaged by IF for KSHV K8 (green), RFP-LANA (red), and DAPI (blue). Scale bar = 10 μM.
(TIF)

S1 Movie Untreated iSLK, DNA (green), LANA (red) 63X6Xpos01_3Drotation Cell. (MP4)

S2 Movie Untreated iSLK, DNA (green), LANA (red) 63X6Xpos01_3Drotation, Crop. (MP4)

S3 Movie Untreated iSLK, DNA (green), DAXX (red) Ctr_D515 Pos04_3Drotation_Cell. (MP4)

S4 Movie Untreated iSLK, DNA (green), DAXX (red) Ctr D515 Pos04_3Drotation_Crop. (MP4)

S5 Movie DOXY_DNA (green)_Lana (red)_63X6Xpos06_3Drotation_Cell. (MP4)

S6 Movie DOXY_DNA (green)_Lana (red)_63X6Xpos06_3Drotation_Crop. (MP4)

S7 Movie DOXY_DNA (green)_DAXX (red)_63X6Xpos03_3Drotation_Cell. (MP4)

S8 Movie DOXY_DNA (green)_DAXX (red)_63X6Xpos03_3Drotation_Crop. (MP4)

## References

[1] Cesarman E, Damania B, Krown SE, Martin J, Bower M, Whitby D. Kaposi sarcoma. Nat Rev Dis Primers. 2019;5(1):9. Epub 2019/02/02. 10.1038/s41572-019-0060-9 . 30705286 PMC6685213

[2] Chang Y, Moore P. Twenty years of KSHV. Viruses. 2014;6(11):4258–64. Epub 2014/11/12. 10.3390/v6114258 . 25386844 PMC4246220

[3] De Leo A, Calderon A, Lieberman PM. Control of Viral Latency by Episome Maintenance Proteins. Trends Microbiol. 2020;28(2):150–62. Epub 2019/10/19. 10.1016/j.tim.2019.09.002 . 31624007 PMC6980450

[4] Ballestas ME, Kaye KM. The latency-associated nuclear antigen, a multifunctional protein central to Kaposi’s sarcoma-associated herpesvirus latency. Future Microbiol. 2011;6(12):1399–413. Epub 2011/11/30. 10.2217/fmb.11.137 . 22122438 PMC3857968

[5] Uppal T, Jha HC, Verma SC, Robertson ES. Chromatinization of the KSHV Genome During the KSHV Life Cycle. Cancers (Basel). 2015;7(1):112–42. Epub 2015/01/17. 10.3390/cancers7010112 . 25594667 PMC4381254

[6] Campbell M, Yang WS, Yeh WW, Kao CH, Chang PC. Epigenetic Regulation of Kaposi’s Sarcoma-Associated Herpesvirus Latency. Front Microbiol. 2020;11:850. Epub 2020/06/09. 10.3389/fmicb.2020.00850 . 32508765 PMC7248258

[7] Frohlich J, Grundhoff A. Epigenetic control in Kaposi sarcoma-associated herpesvirus infection and associated disease. Semin Immunopathol. 2020;42(2):143–57. Epub 2020/03/29. 10.1007/s00281-020-00787-z . 32219477 PMC7174275

[8] Uppal T, Banerjee S, Sun Z, Verma SC, Robertson ES. KSHV LANA—the master regulator of KSHV latency. Viruses. 2014;6(12):4961–98. Epub 2014/12/17. 10.3390/v6124961 . 25514370 PMC4276939

[9] Garber AC, Shu MA, Hu J, Renne R. DNA binding and modulation of gene expression by the latency-associated nuclear antigen of Kaposi’s sarcoma-associated herpesvirus. J Virol. 2001;75(17):7882–92. Epub 2001/08/03. 10.1128/jvi.75.17.7882-7892.2001 . 11483733 PMC115032

[10] Hellert J, Weidner-Glunde M, Krausze J, Lunsdorf H, Ritter C, Schulz TF, et al. The 3D structure of Kaposi sarcoma herpesvirus LANA C-terminal domain bound to DNA. Proc Natl Acad Sci U S A. 2015;112(21):6694–9. Epub 2015/05/08. 10.1073/pnas.1421804112 . 25947153 PMC4450395

[11] Ballestas ME, Chatis PA, Kaye KM. Efficient persistence of extrachromosomal KSHV DNA mediated by latency-associated nuclear antigen. Science. 1999;284(5414):641–4. Epub 1999/04/24. 10.1126/science.284.5414.641 . 10213686

[12] Hu J, Yang Y, Turner PC, Jain V, McIntyre LM, Renne R. LANA binds to multiple active viral and cellular promoters and associates with the H3K4methyltransferase hSET1 complex. PLoS Pathog. 2014;10(7):e1004240. Epub 2014/07/18. 10.1371/journal.ppat.1004240 . 25033463 PMC4102568

[13] Mercier A, Arias C, Madrid AS, Holdorf MM, Ganem D. Site-specific association with host and viral chromatin by Kaposi’s sarcoma-associated herpesvirus LANA and its reversal during lytic reactivation. J Virol. 2014;88(12):6762–77. Epub 2014/04/04. 10.1128/JVI.00268-14 . 24696474 PMC4054346

[14] Lu F, Tsai K, Chen HS, Wikramasinghe P, Davuluri RV, Showe L, et al. Identification of host-chromosome binding sites and candidate gene targets for Kaposi’s sarcoma-associated herpesvirus LANA. J Virol. 2012;86(10):5752–62. Epub 2012/03/16. 10.1128/JVI.07216-11 . 22419807 PMC3347294

[15] Sun R, Tan X, Wang X, Wang X, Yang L, Robertson ES, et al. Epigenetic Landscape of Kaposi’s Sarcoma-Associated Herpesvirus Genome in Classic Kaposi’s Sarcoma Tissues. PLoS Pathog. 2017;13(1):e1006167. Epub 2017/01/25. 10.1371/journal.ppat.1006167 . 28118409 PMC5291540

[16] Lan K, Kuppers DA, Verma SC, Robertson ES. Kaposi’s sarcoma-associated herpesvirus-encoded latency-associated nuclear antigen inhibits lytic replication by targeting Rta: a potential mechanism for virus-mediated control of latency. J Virol. 2004;78(12):6585–94. Epub 2004/05/28. 10.1128/JVI.78.12.6585-6594.2004 . 15163750 PMC416549

[17] Lu F, Day L, Gao SJ, Lieberman PM. Acetylation of the latency-associated nuclear antigen regulates repression of Kaposi’s sarcoma-associated herpesvirus lytic transcription. J Virol. 2006;80(11):5273–82. Epub 2006/05/16. 10.1128/JVI.02541-05 . 16699007 PMC1472144

[18] De Leo A, Deng Z, Vladimirova O, Chen HS, Dheekollu J, Calderon A, et al. LANA oligomeric architecture is essential for KSHV nuclear body formation and viral genome maintenance during latency. PLoS Pathog. 2019;15(1):e1007489. Epub 2019/01/27. 10.1371/journal.ppat.1007489 . 30682185 PMC6364946

[19] Hellert J, Weidner-Glunde M, Krausze J, Richter U, Adler H, Fedorov R, et al. A structural basis for BRD2/4-mediated host chromatin interaction and oligomer assembly of Kaposi sarcoma-associated herpesvirus and murine gammaherpesvirus LANA proteins. PLoS Pathog. 2013;9(10):e1003640. Epub 2013/10/23. 10.1371/journal.ppat.1003640 . 24146614 PMC3798688

[20] Domsic JF, Chen HS, Lu F, Marmorstein R, Lieberman PM. Molecular basis for oligomeric-DNA binding and episome maintenance by KSHV LANA. PLoS Pathog. 2013;9(10):e1003672. Epub 2013/10/23. 10.1371/journal.ppat.1003672 . 24146617 PMC3798644

[21] Barbera AJ, Chodaparambil JV, Kelley-Clarke B, Joukov V, Walter JC, Luger K, et al. The nucleosomal surface as a docking station for Kaposi’s sarcoma herpesvirus LANA. Science. 2006;311(5762):856–61. Epub 2006/02/14. 10.1126/science.1120541 . 16469929

[22] Ueda K. KSHV Genome Replication and Maintenance in Latency. Adv Exp Med Biol. 2018;1045:299–320. Epub 2018/06/14. 10.1007/978-981-10-7230-7_14 . 29896673

[23] Weidner-Glunde M, Mariggio G, Schulz TF. Kaposi’s Sarcoma-Associated Herpesvirus Latency-Associated Nuclear Antigen: Replicating and Shielding Viral DNA during Viral Persistence. J Virol. 2017;91(14). Epub 2017/04/28. 10.1128/JVI.01083-16 . 28446671 PMC5487577

[24] Cohan MC, Pappu RV. Making the Case for Disordered Proteins and Biomolecular Condensates in Bacteria. Trends Biochem Sci. 2020. Epub 2020/05/28. 10.1016/j.tibs.2020.04.011 . 32456986

[25] Dignon GL, Best RB, Mittal J. Biomolecular Phase Separation: From Molecular Driving Forces to Macroscopic Properties. Annu Rev Phys Chem. 2020;71:53–75. Epub 2020/04/22. 10.1146/annurev-physchem-071819-113553 . 32312191 PMC7469089

[26] Sehgal PB, Westley J, Lerea KM, DiSenso-Browne S, Etlinger JD. Biomolecular condensates in cell biology and virology: Phase-separated membraneless organelles (MLOs). Anal Biochem. 2020;597:113691. Epub 2020/03/21. 10.1016/j.ab.2020.113691 . 32194074

[27] Sanulli S, Trnka MJ, Dharmarajan V, Tibble RW, Pascal BD, Burlingame AL, et al. HP1 reshapes nucleosome core to promote phase separation of heterochromatin. Nature. 2019;575(7782):390–4. Epub 2019/10/17. 10.1038/s41586-019-1669-2 . 31618757 PMC7039410

[28] Hnisz D, Shrinivas K, Young RA, Chakraborty AK, Sharp PA. A Phase Separation Model for Transcriptional Control. Cell. 2017;169(1):13–23. Epub 2017/03/25. 10.1016/j.cell.2017.02.007 . 28340338 PMC5432200

[29] Sabari BR, Dall’Agnese A, Boija A, Klein IA, Coffey EL, Shrinivas K, et al. Coactivator condensation at super-enhancers links phase separation and gene control. Science. 2018;361(6400). Epub 2018/06/23. 10.1126/science.aar3958 . 29930091 PMC6092193

[30] Guo YE, Manteiga JC, Henninger JE, Sabari BR, Dall’Agnese A, Hannett NM, et al. Pol II phosphorylation regulates a switch between transcriptional and splicing condensates. Nature. 2019;572(7770):543–8. Epub 2019/08/09. 10.1038/s41586-019-1464-0 . 31391587 PMC6706314

[31] Soding J, Zwicker D, Sohrabi-Jahromi S, Boehning M, Kirschbaum J. Mechanisms for Active Regulation of Biomolecular Condensates. Trends Cell Biol. 2020;30(1):4–14. Epub 2019/11/23. 10.1016/j.tcb.2019.10.006 . 31753533

[32] Sleeman JE, Trinkle-Mulcahy L, Prescott AR, Ogg SC, Lamond AI. Cajal body proteins SMN and Coilin show differential dynamic behaviour in vivo. J Cell Sci. 2003;116(Pt 10):2039–50. Epub 2003/04/08. 10.1242/jcs.00400 . 12679382

[33] Kato M, McKnight SL. A Solid-State Conceptualization of Information Transfer from Gene to Message to Protein. Annu Rev Biochem. 2018;87:351–90. Epub 2017/12/02. 10.1146/annurev-biochem-061516-044700 . 29195049

[34] Kobiler O, Brodersen P, Taylor MP, Ludmir EB, Enquist LW. Herpesvirus replication compartments originate with single incoming viral genomes. mBio. 2011;2(6). Epub 2011/12/22. 10.1128/mBio.00278-11 . 22186611 PMC3269065

[35] Kobiler O, Lipman Y, Therkelsen K, Daubechies I, Enquist LW. Herpesviruses carrying a Brainbow cassette reveal replication and expression of limited numbers of incoming genomes. Nat Commun. 2010;1:146. Epub 2011/01/27. 10.1038/ncomms1145 . 21266996 PMC3079281

[36] Charman M, Weitzman MD. Replication Compartments of DNA Viruses in the Nucleus: Location, Location, Location. Viruses. 2020;12(2). Epub 2020/02/06. 10.3390/v12020151 . 32013091 PMC7077188

[37] Sawyer IA, Hager GL, Dundr M. Specific genomic cues regulate Cajal body assembly. RNA Biol. 2017;14(6):791–803. Epub 2016/10/08. 10.1080/15476286.2016.1243648 . 27715441 PMC5519236

[38] Kang H, Wiedmer A, Yuan Y, Robertson E, Lieberman PM. Coordination of KSHV latent and lytic gene control by CTCF-cohesin mediated chromosome conformation. PLoS Pathog. 2011;7(8):e1002140. Epub 2011/08/31. 10.1371/journal.ppat.1002140 . 21876668 PMC3158054

[39] Chen HS, De Leo A, Wang Z, Kerekovic A, Hills R, Lieberman PM. BET-Inhibitors Disrupt Rad21-Dependent Conformational Control of KSHV Latency. PLoS Pathog. 2017;13(1):e1006100. Epub 2017/01/21. 10.1371/journal.ppat.1006100 . 28107481 PMC5287475

[40] Gunther T, Schreiner S, Dobner T, Tessmer U, Grundhoff A. Influence of ND10 components on epigenetic determinants of early KSHV latency establishment. PLoS Pathog. 2014;10(7):e1004274. Epub 2014/07/18. 10.1371/journal.ppat.1004274 . 25033267 PMC4102598

[41] Shin Y, Brangwynne CP. Liquid phase condensation in cell physiology and disease. Science. 2017;357(6357). Epub 2017/09/25. 10.1126/science.aaf4382 . 28935776

[42] Banani SF, Lee HO, Hyman AA, Rosen MK. Biomolecular condensates: organizers of cellular biochemistry. Nat Rev Mol Cell Biol. 2017;18(5):285–98. Epub 2017/02/23. 10.1038/nrm.2017.7 . 28225081 PMC7434221

[43] Hyman AA, Weber CA, Julicher F. Liquid-liquid phase separation in biology. Annu Rev Cell Dev Biol. 2014;30:39–58. Epub 2014/10/08. 10.1146/annurev-cellbio-100913-013325 . 25288112

[44] Charman M, Herrmann C, Weitzman MD. Viral and cellular interactions during adenovirus DNA replication. FEBS Lett. 2019;593(24):3531–50. Epub 2019/11/26. 10.1002/1873-3468.13695 . 31764999 PMC6928415

[45] Taylor TJ, McNamee EE, Day C, Knipe DM. Herpes simplex virus replication compartments can form by coalescence of smaller compartments. Virology. 2003;309(2):232–47. Epub 2003/05/22. 10.1016/s0042-6822(03)00107-7 . 12758171

[46] Li Z, Fang C, Su Y, Liu H, Lang F, Li X, et al. Visualizing the replicating HSV-1 virus using STED super-resolution microscopy. Virol J. 2016;13:65. Epub 2016/04/12. 10.1186/s12985-016-0521-7 . 27062411 PMC4826541

[47] Strang BL, Boulant S, Chang L, Knipe DM, Kirchhausen T, Coen DM. Human cytomegalovirus UL44 concentrates at the periphery of replication compartments, the site of viral DNA synthesis. J Virol. 2012;86(4):2089–95. Epub 2011/12/14. 10.1128/JVI.06720-11 . 22156516 PMC3302373

[48] Vallery TK, Withers JB, Andoh JA, Steitz JA. Kaposi’s Sarcoma-Associated Herpesvirus mRNA Accumulation in Nuclear Foci Is Influenced by Viral DNA Replication and Viral Noncoding Polyadenylated Nuclear RNA. J Virol. 2018;92(13). Epub 2018/04/13. 10.1128/JVI.00220-18 . 29643239 PMC6002709

[49] Livingston CM, Ifrim MF, Cowan AE, Weller SK. Virus-Induced Chaperone-Enriched (VICE) domains function as nuclear protein quality control centers during HSV-1 infection. PLoS Pathog. 2009;5(10):e1000619. Epub 2009/10/10. 10.1371/journal.ppat.1000619 . 19816571 PMC2752995

[50] Mitrea DM, Kriwacki RW. Phase separation in biology; functional organization of a higher order. Cell Commun Signal. 2016;14:1. Epub 2016/01/06. 10.1186/s12964-015-0125-7 . 26727894 PMC4700675

[51] Roden C, Gladfelter AS. RNA contributions to the form and function of biomolecular condensates. Nat Rev Mol Cell Biol. 2020. Epub 2020/07/08. 10.1038/s41580-020-0264-6 . 32632317 PMC7785677

[52] Kelley-Clarke B, Ballestas ME, Komatsu T, Kaye KM. Kaposi’s sarcoma herpesvirus C-terminal LANA concentrates at pericentromeric and peri-telomeric regions of a subset of mitotic chromosomes. Virology. 2007;357(2):149–57. Epub 2006/09/19. 10.1016/j.virol.2006.07.052 . 16979209

[53] Cai QL, Knight JS, Verma SC, Zald P, Robertson ES. EC5S ubiquitin complex is recruited by KSHV latent antigen LANA for degradation of the VHL and p53 tumor suppressors. PLoS Pathog. 2006;2(10):e116. Epub 2006/10/31. 10.1371/journal.ppat.0020116 . 17069461 PMC1626105

[54] Cai Q, Cai S, Zhu C, Verma SC, Choi JY, Robertson ES. A unique SUMO-2-interacting motif within LANA is essential for KSHV latency. PLoS Pathog. 2013;9(11):e1003750. Epub 2013/11/28. 10.1371/journal.ppat.1003750 . 24278015 PMC3836728

[55] Murakami Y, Yamagoe S, Noguchi K, Takebe Y, Takahashi N, Uehara Y, et al. Ets-1-dependent expression of vascular endothelial growth factor receptors is activated by latency-associated nuclear antigen of Kaposi’s sarcoma-associated herpesvirus through interaction with Daxx. J Biol Chem. 2006;281(38):28113–21. Epub 2006/07/25. 10.1074/jbc.M602026200 . 16861237

[56] Lim C, Gwack Y, Hwang S, Kim S, Choe J. The transcriptional activity of cAMP response element-binding protein-binding protein is modulated by the latency associated nuclear antigen of Kaposi’s sarcoma-associated herpesvirus. J Biol Chem. 2001;276(33):31016–22. Epub 2001/06/27. 10.1074/jbc.M102431200 . 11425857

[57] Hollingworth R, Horniblow RD, Forrest C, Stewart GS, Grand RJ. Localization of Double-Strand Break Repair Proteins to Viral Replication Compartments following Lytic Reactivation of Kaposi’s Sarcoma-Associated Herpesvirus. J Virol. 2017;91(22). Epub 2017/09/01. 10.1128/JVI.00930-17 . 28855246 PMC5660498

[58] Vallery TK, Steitz JA. Quantitative Fluorescence In Situ Hybridization (FISH) and Immunofluorescence (IF) of Specific Gene Products in KSHV-Infected Cells. J Vis Exp. 2019;(150). Epub 2019/09/17. 10.3791/59697 . 31524859 PMC6750728

[59] Gardner MR, Glaunsinger BA. Kaposi’s Sarcoma-Associated Herpesvirus ORF68 Is a DNA Binding Protein Required for Viral Genome Cleavage and Packaging. J Virol. 2018;92(16). Epub 2018/06/08. 10.1128/JVI.00840-18 . 29875246 PMC6069193

[60] Garrigues HJ, Howard K, Barcy S, Ikoma M, Moses AV, Deutsch GH, et al. Full-Length Isoforms of Kaposi’s Sarcoma-Associated Herpesvirus Latency-Associated Nuclear Antigen Accumulate in the Cytoplasm of Cells Undergoing the Lytic Cycle of Replication. J Virol. 2017;91(24). Epub 2017/10/06. 10.1128/JVI.01532-17 . 28978712 PMC5709576

[61] Wu FY, Ahn JH, Alcendor DJ, Jang WJ, Xiao J, Hayward SD, et al. Origin-independent assembly of Kaposi’s sarcoma-associated herpesvirus DNA replication compartments in transient cotransfection assays and association with the ORF-K8 protein and cellular PML. J Virol. 2001;75(3):1487–506. Epub 2001/01/11. 10.1128/JVI.75.3.1487-1506.2001 . 11152521 PMC114054

[62] Elsasser SJ, Huang H, Lewis PW, Chin JW, Allis CD, Patel DJ. DAXX envelops a histone H3.3-H4 dimer for H3.3-specific recognition. Nature. 2012;491(7425):560–5. Epub 2012/10/19. 10.1038/nature11608 . 23075851 PMC4056191

[63] Shih HM, Chang CC, Kuo HY, Lin DY. Daxx mediates SUMO-dependent transcriptional control and subnuclear compartmentalization. Biochem Soc Trans. 2007;35(Pt 6):1397–400. Epub 2007/11/23. 10.1042/BST0351397 . 18031230

[64] Guseva S, Milles S, Jensen MR, Salvi N, Kleman JP, Maurin D, et al. Measles virus nucleo- and phosphoproteins form liquid-like phase-separated compartments that promote nucleocapsid assembly. Sci Adv. 2020;6(14):eaaz7095. Epub 2020/04/10. 10.1126/sciadv.aaz7095 . 32270045 PMC7112944

[65] Heinrich BS, Maliga Z, Stein DA, Hyman AA, Whelan SPJ. Phase Transitions Drive the Formation of Vesicular Stomatitis Virus Replication Compartments. mBio. 2018;9(5). Epub 2018/09/06. 10.1128/mBio.02290-17 . 30181255 PMC6123442

[66] Peng Q, Wang L, Qin Z, Wang J, Zheng X, Wei L, et al. Phase Separation of Epstein-Barr Virus EBNA2 and Its Coactivator EBNALP Controls Gene Expression. J Virol. 2020;94(7). Epub 2020/01/17. 10.1128/JVI.01771-19 . 31941785 PMC7081900

[67] McSwiggen DT, Hansen AS, Teves SS, Marie-Nelly H, Hao Y, Heckert AB, et al. Evidence for DNA-mediated nuclear compartmentalization distinct from phase separation. Elife. 2019;8. Epub 2019/05/01. 10.7554/eLife.47098 . 31038454 PMC6522219

[68] Alberti S, Gladfelter A, Mittag T. Considerations and Challenges in Studying Liquid-Liquid Phase Separation and Biomolecular Condensates. Cell. 2019;176(3):419–34. Epub 2019/01/27. 10.1016/j.cell.2018.12.035 . 30682370 PMC6445271

[69] Myoung J, Ganem D. Generation of a doxycycline-inducible KSHV producer cell line of endothelial origin: maintenance of tight latency with efficient reactivation upon induction. J Virol Methods. 2011;174(1–2):12–21. Epub 2011/03/23. 10.1016/j.jviromet.2011.03.012 . 21419799 PMC3095772

[70] Renne R, Zhong W, Herndier B, McGrath M, Abbey N, Kedes D, et al. Lytic growth of Kaposi’s sarcoma-associated herpesvirus (human herpesvirus 8) in culture. Nat Med. 1996;2(3):342–6. Epub 1996/03/01. 10.1038/nm0396-342 . 8612236

[71] Brulois KF, Chang H, Lee AS, Ensser A, Wong LY, Toth Z, et al. Construction and manipulation of a new Kaposi’s sarcoma-associated herpesvirus bacterial artificial chromosome clone. J Virol. 2012;86(18):9708–20. Epub 2012/06/29. 10.1128/JVI.01019-12 . 22740391 PMC3446615

[72] Huang H, Deng Z, Vladimirova O, Wiedmer A, Lu F, Lieberman PM, et al. Structural basis underlying viral hijacking of a histone chaperone complex. Nat Commun. 2016;7:12707. Epub 2016/09/02. 10.1038/ncomms12707 . 27581705 PMC5025803

[73] Deng Z, Wang Z, Stong N, Plasschaert R, Moczan A, Chen HS, et al. A role for CTCF and cohesin in subtelomere chromatin organization, TERRA transcription, and telomere end protection. EMBO J. 2012;31(21):4165–78. Epub 2012/09/27. 10.1038/emboj.2012.266 . 23010778 PMC3492729

