# Supplementary figures and images for: Phase separation and DAXX redistribution contribute to LANA nuclear body and KSHV genome dynamics during latency and reactivation
Source: PLoS Pathog. 2021 Jan 20;17(1):e1009231. doi: 10.1371/journal.ppat.1009231 (PMC7943007; doi:10.1371/journal.ppat.1009231)

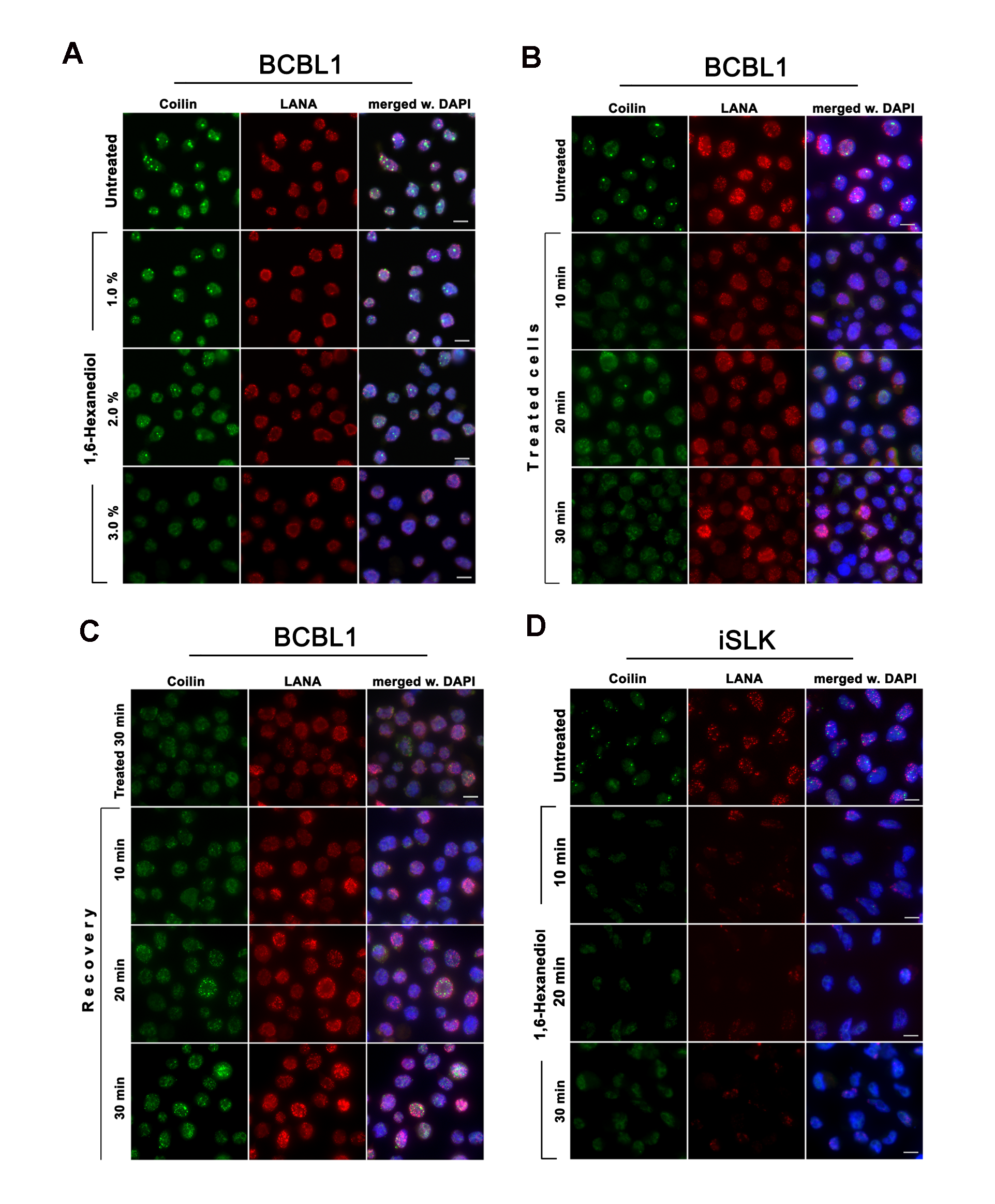

Supplement: S1 Fig — A. BCBL1 cells were untreated or treated with 1.0, 2.0, or 3.0% 1,6-HD for 15 min and imaged by IF for Coilin(green) or LANA (red) or merged with DAPI (blue). Scale bar = 10 μm. B. BCBL1 cells were treated with 3.5% 1,6-Hexanediol for either 10, 20, or 30 min and imaged by IF as in panel A. Scale bar = 10 μm. C. BCBL1 cells were treated for 30 min with 3.5% 1,6 Hexanediol (top panel) followed by recovery in fresh media for 10, 20, or 30 min and imaged by IF as in panel A. Scale bar = 10 μm. D. iSLK cells were treated with 3.5% 1,6-HD for 10, 20, or 30 min and imaged by IF as in panel A. Scale bar = 10 μm. (TIF) [file ppat.1009231.s001.tif]

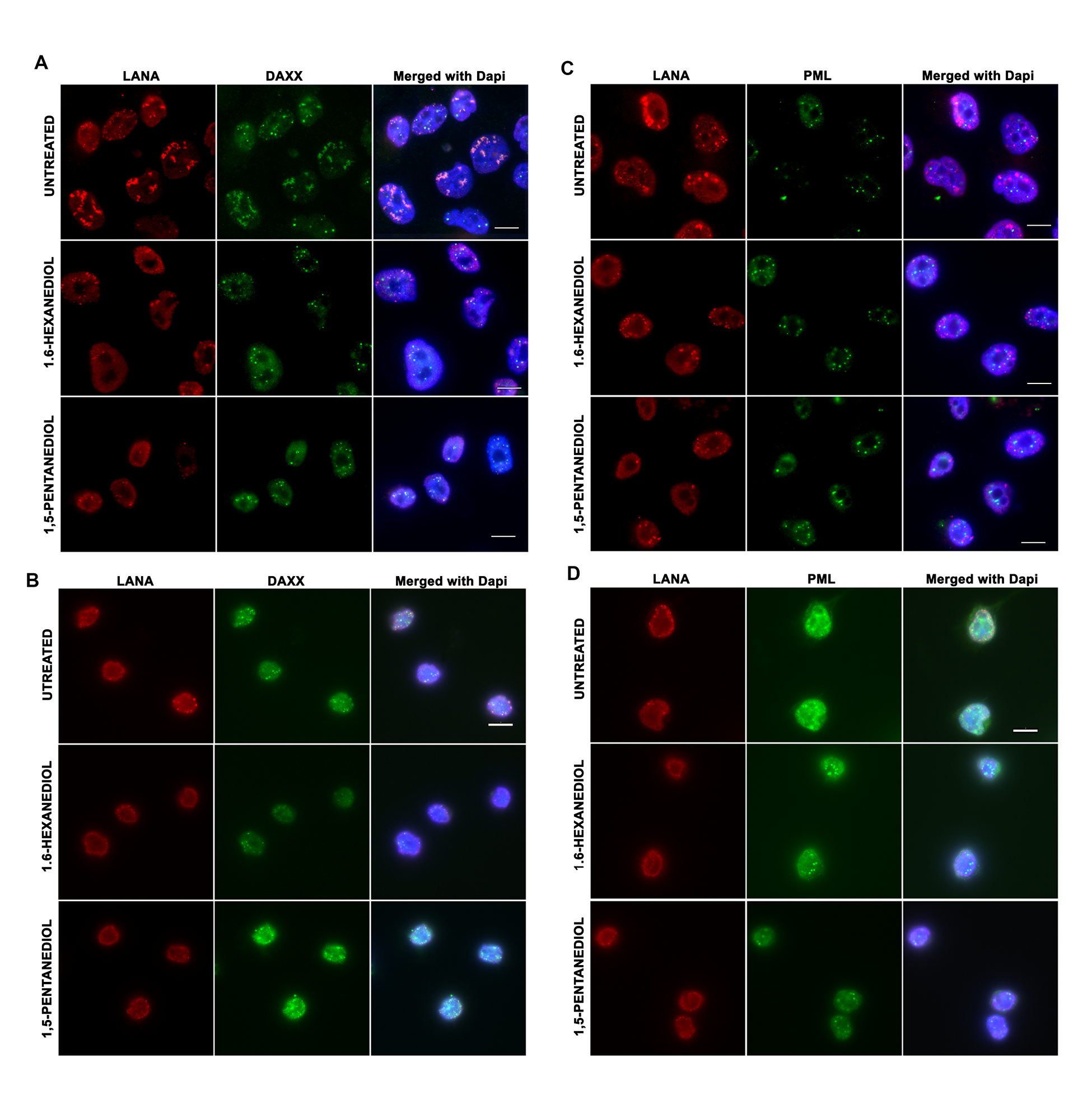

Supplement: S2 Fig — A. iSLK RFP-LANA cells were either untreated or treated with 3.5% 1,6-HD or 1,5-PD for 10 min and then assayed by IF for LANA (red) or DAXX (green) or merged with DAPI (blue). B. BCBL1 cells were treated same as in panel A, except IF with LANA (red), and DAXX (green). C. iSLK RFP-LANA cells treated as in panel A, but IF with PML (green) and merged with DAPI (blue). D. BCBL1 cells treated as in panel C, except IF with LANA (red) and PML (green). Scale bar = 10μm. (TIF) [file ppat.1009231.s002.tif]

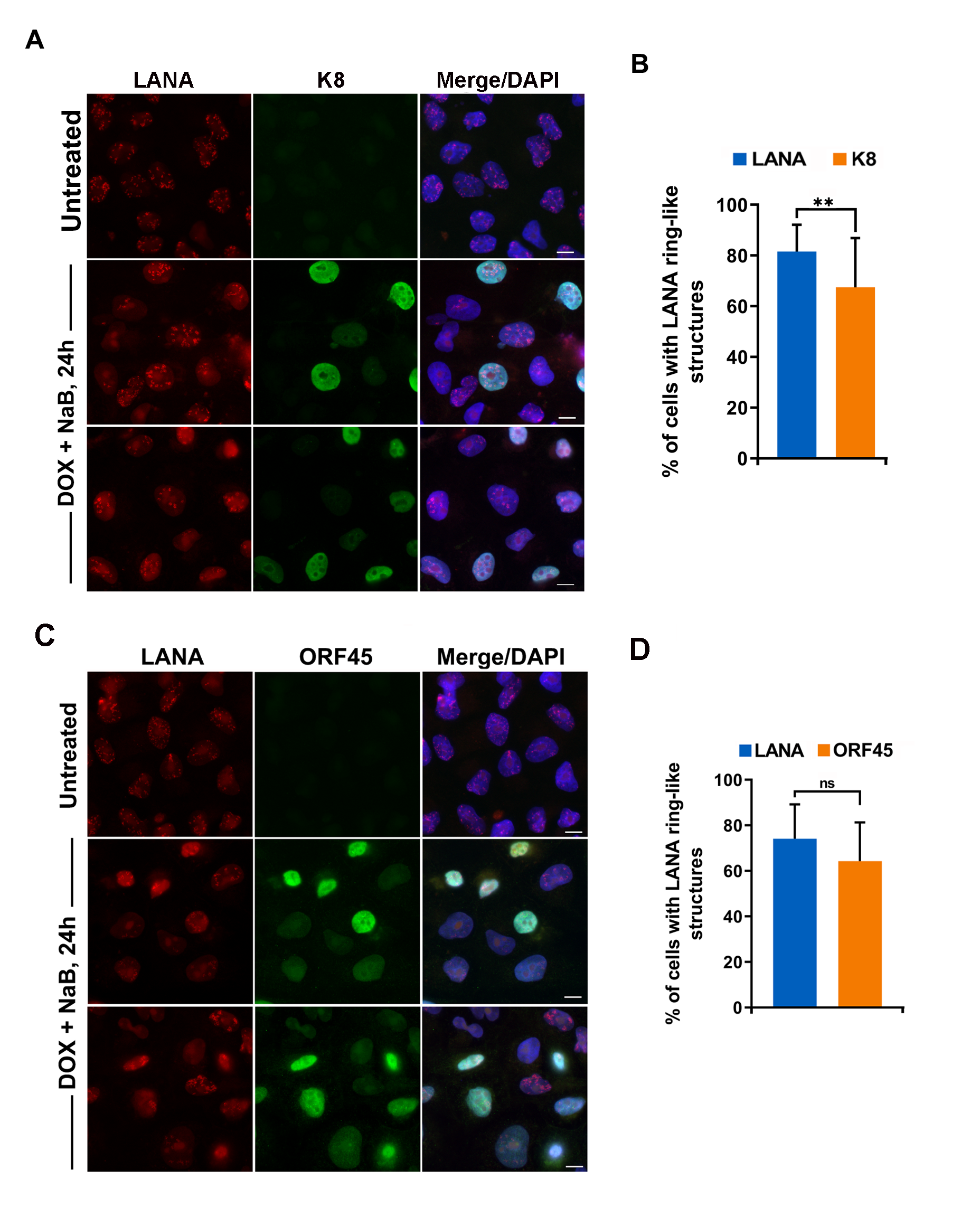

Supplement: S3 Fig — A. iSLK RFP-LANA cells were either untreated or induced with Dox+NaB for 24 h and imaged by IF for K8 (green), RFP-LANA (red), and DAPI (blue). Scale bar = 10μM. B. Quantification of cell images represented in panel A, for percent of cells with LANA ring-like structures for all LANA positive cells (blue) or K8 positive cells (yellow). The bar graphs represent mean± s.d., p value not significant (ns), two tailed t-test, relative to total LANA rings. C. Same as in panel A, except with ORF45 (green). D. Quantification for panel C, as described in B. (TIF) [file ppat.1009231.s003.tif]

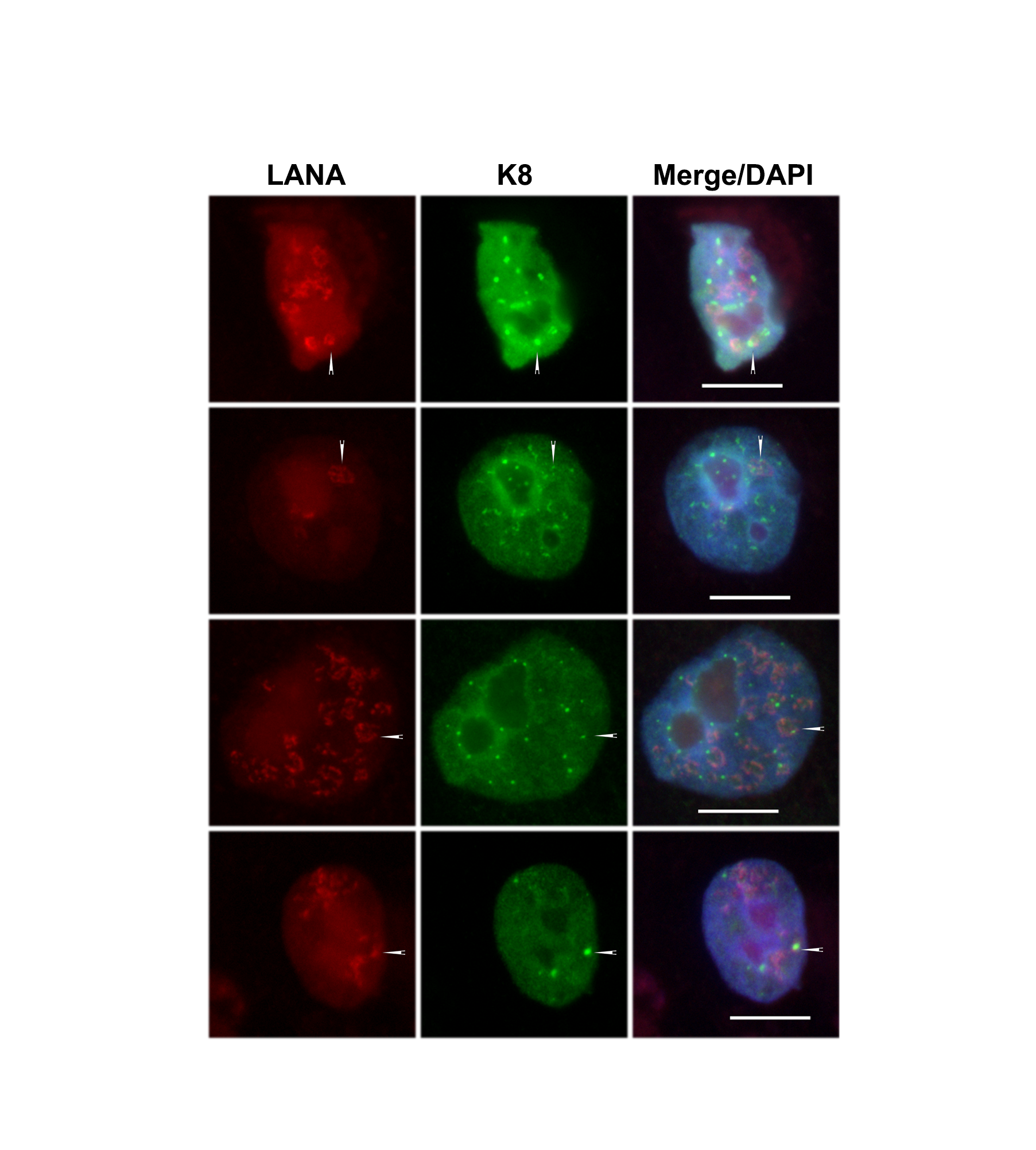

Supplement: S4 Fig — iSLK RFP-LANA cells were induced with DOX+NaB for 24 h and imaged by IF for KSHV K8 (green), RFP-LANA (red), and DAPI (blue). Scale bar = 10 μM. (TIF) [file ppat.1009231.s004.tif]
